# Supplementary material for: Impact of Treatment with RUTF on Plasma Lipid Profiles of Severely Malnourished Pakistani Children
Source: Nutrients. 2020 Jul 21;12(7):2163. doi: 10.3390/nu12072163 (PMC7401247; doi:10.3390/nu12072163)
Supplement: Supplementary file 1 [file nutrients-12-02163-s001.zip › Supplementary Material/Supplementary Figure S1.pdf]

Supplementary Figure S1: Polar lipid composition. Median and interquartile ranges shown for baseline (Before) and after three months of treatment with RUTF (After). **Legend:** *p*-values after FDR adjustment are expressed as ***$p<0.001$, ** $p<0.01$, * $p<0.05$, ns non-significant

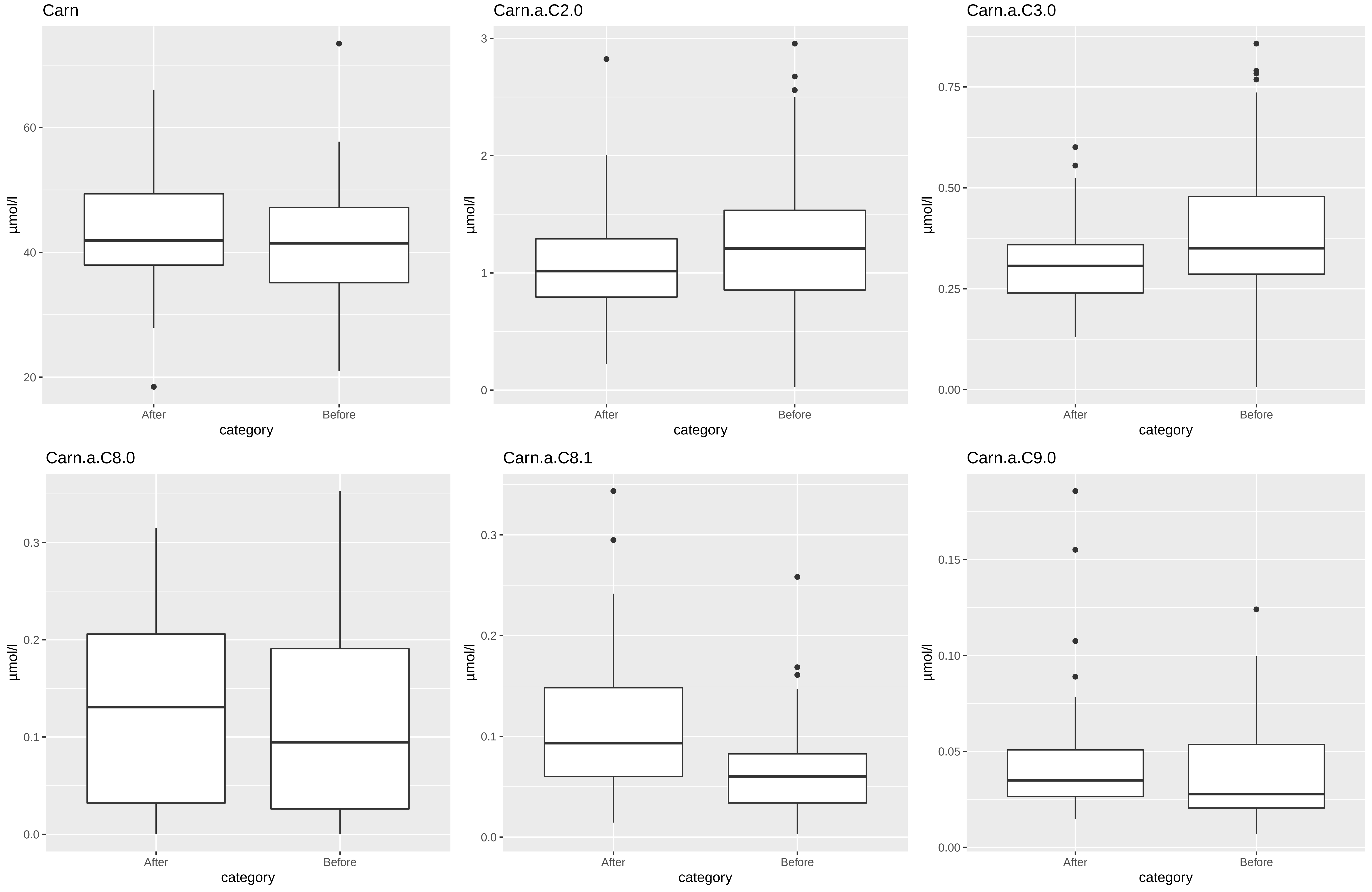
Carn
Carn.a.C2.0
Carn.a.C3.0
Carn.a.C8.0
Carn.a.C8.1
Carn.a.C9.0
µmol/l
category
After
Before
20
40
60
0
1
2
3
0.00
0.25
0.50
0.75
0.0
0.1
0.2
0.3
0.05
0.10
0.15

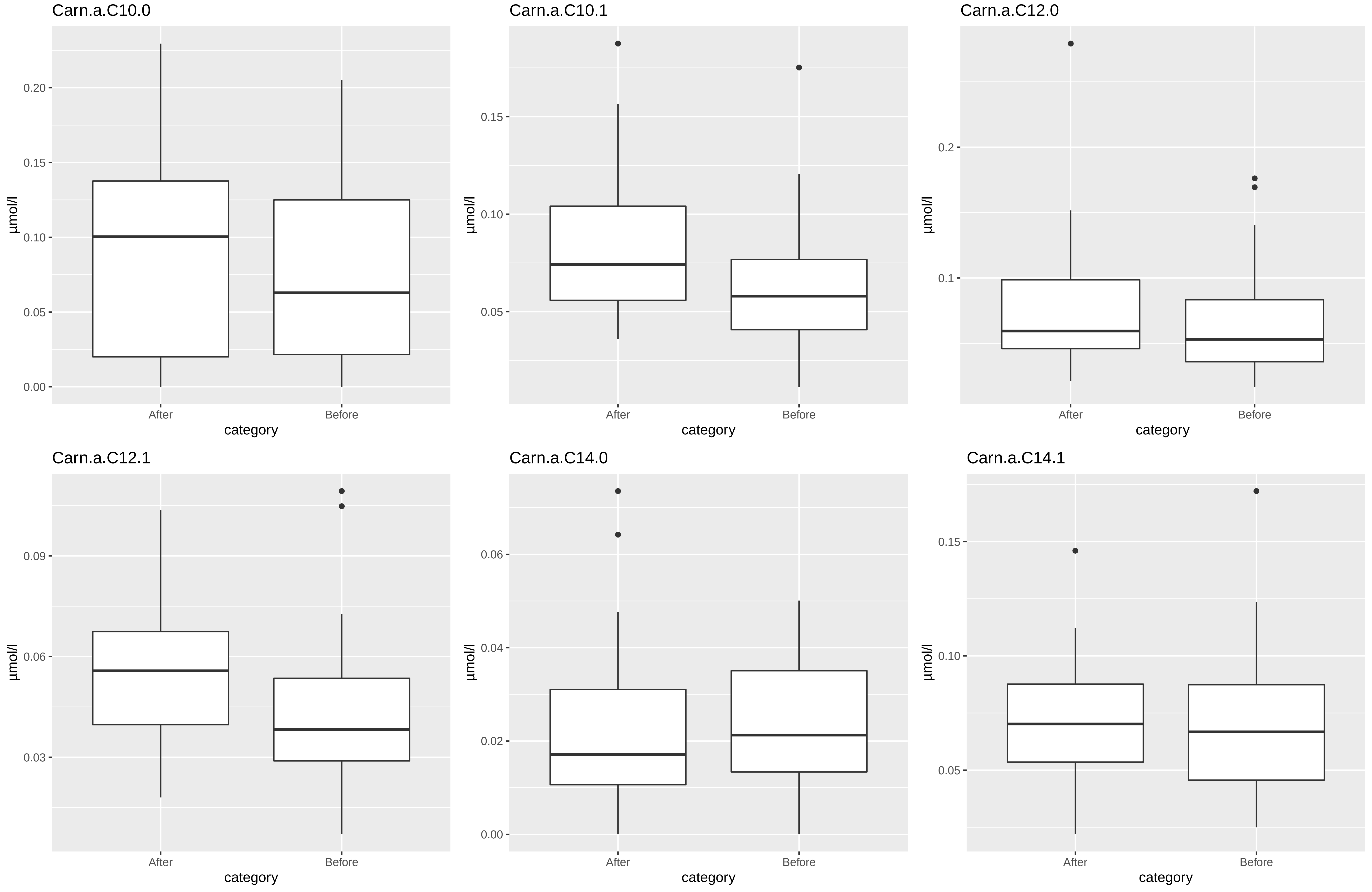
Carn.a.C10.0
0.20
0.15
0.10
0.05
0.00
µmol/l
After
Before
category
Carn.a.C10.1
0.15
0.10
0.05
µmol/l
After
Before
category
Carn.a.C12.0
0.2
0.1
µmol/l
After
Before
category
Carn.a.C12.1
0.09
0.06
0.03
µmol/l
After
Before
category
Carn.a.C14.0
0.06
0.04
0.02
0.00
µmol/l
After
Before
category
Carn.a.C14.1
0.15
0.10
0.05
µmol/l
After
Before
category

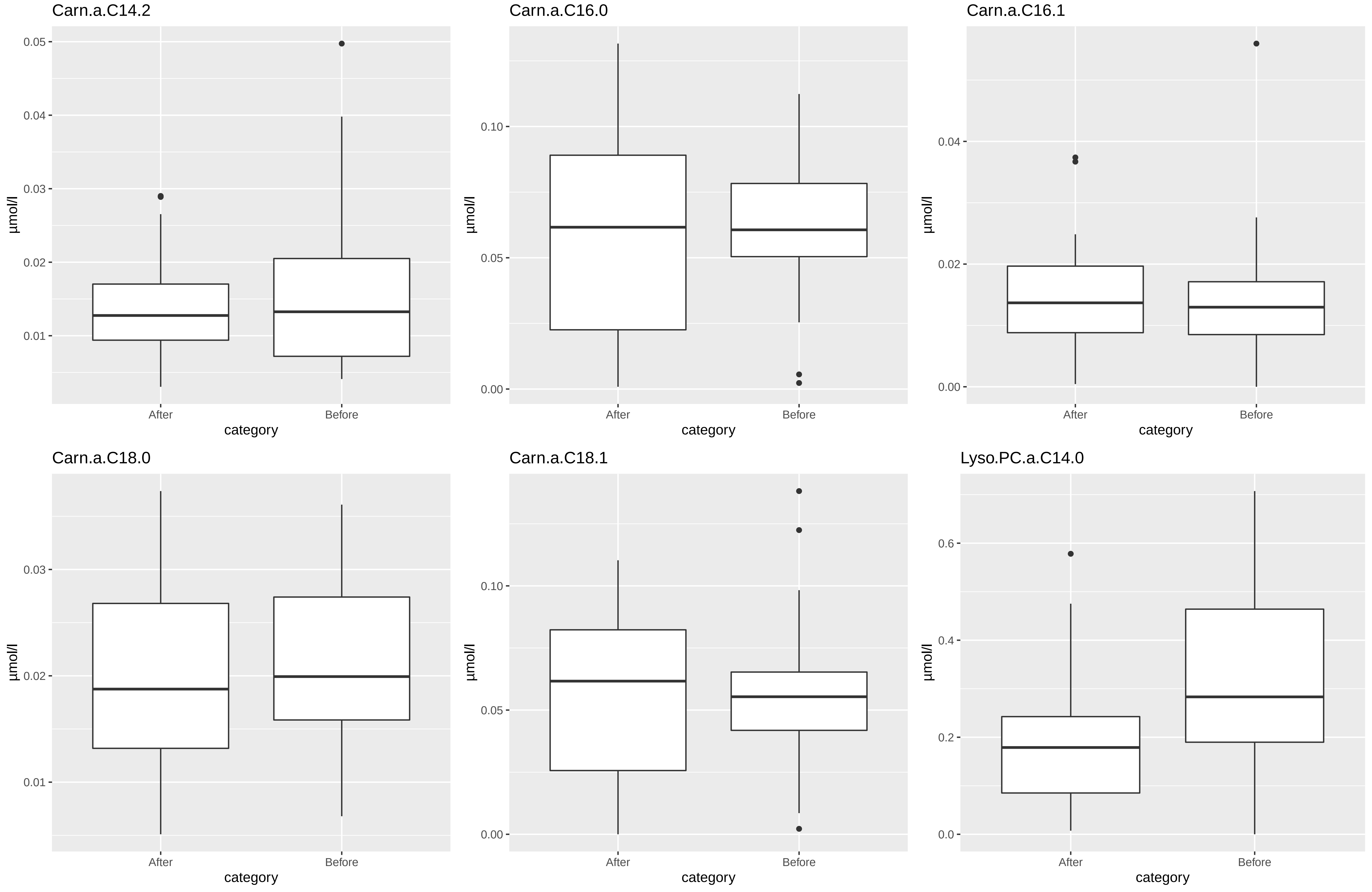
Carn.a.C14.2
Carn.a.C16.0
Carn.a.C16.1
Carn.a.C18.0
Carn.a.C18.1
Lyso.PC.a.C14.0
µmol/l
category
After
Before
0.01
0.02
0.03
0.04
0.05
0.00
0.10
0.2
0.4
0.6
0.0

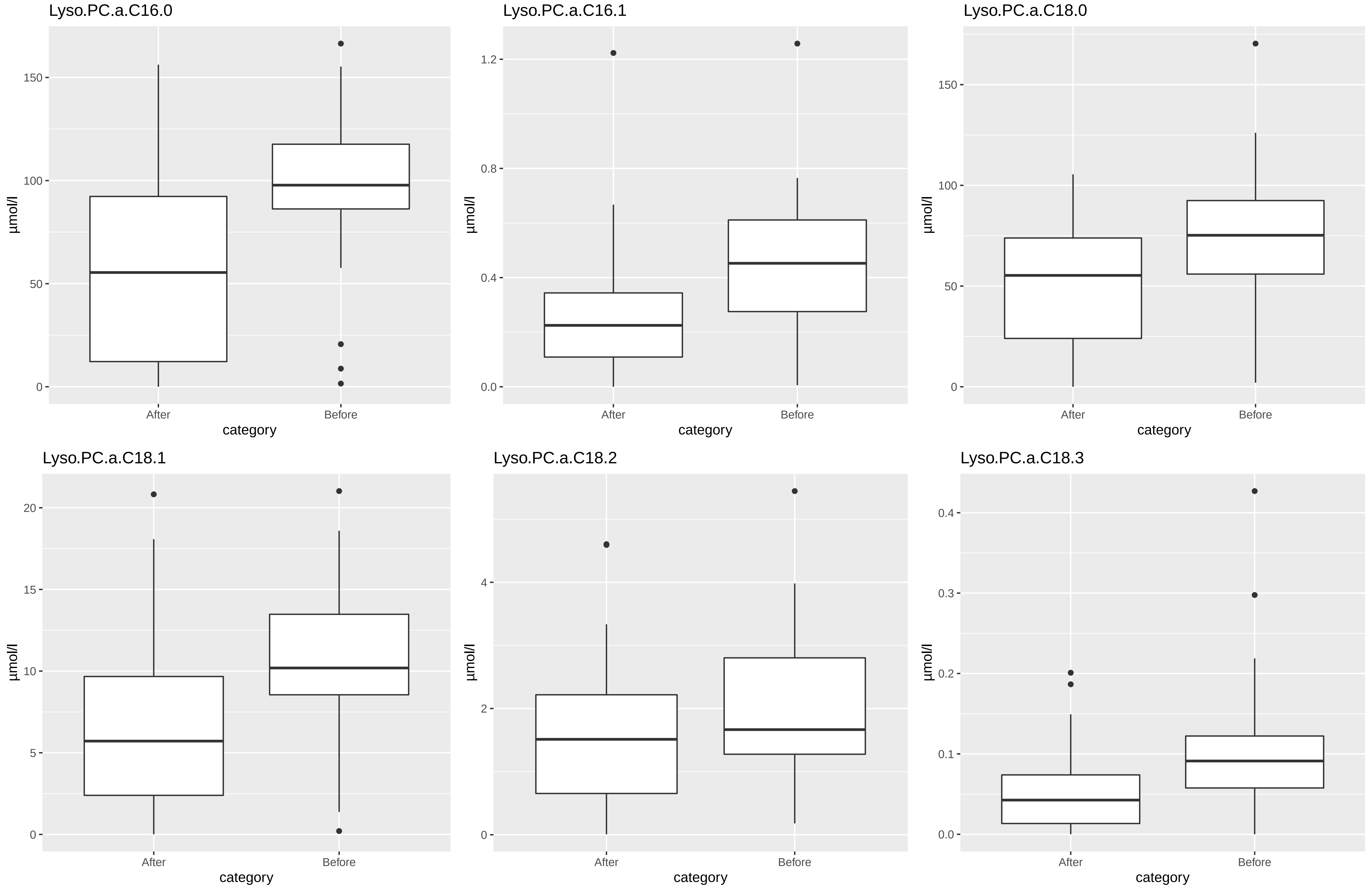
Lyso.PC.a.C16.0
Lyso.PC.a.C16.1
Lyso.PC.a.C18.0
Lyso.PC.a.C18.1
Lyso.PC.a.C18.2
Lyso.PC.a.C18.3
µmol/l
After
Before
category
0
50
100
150
0.0
0.4
0.8
1.2
5
10
15
20
2
4
0.1
0.2
0.3
0.4

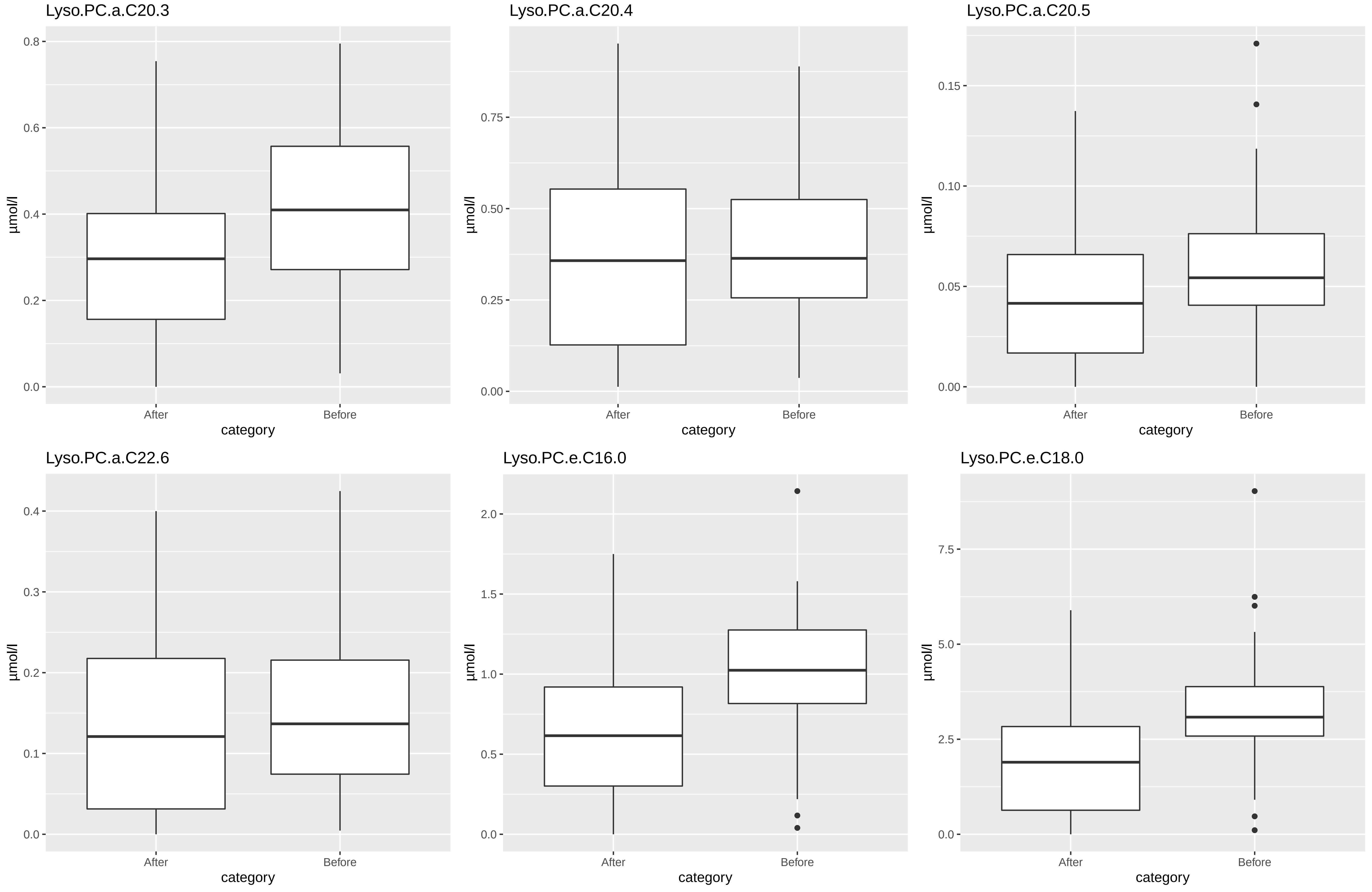

Lyso.PC.a.C20.3
Lyso.PC.a.C20.4
Lyso.PC.a.C20.5
Lyso.PC.a.C22.6
Lyso.PC.e.C16.0
Lyso.PC.e.C18.0
µmol/l
category
After
Before

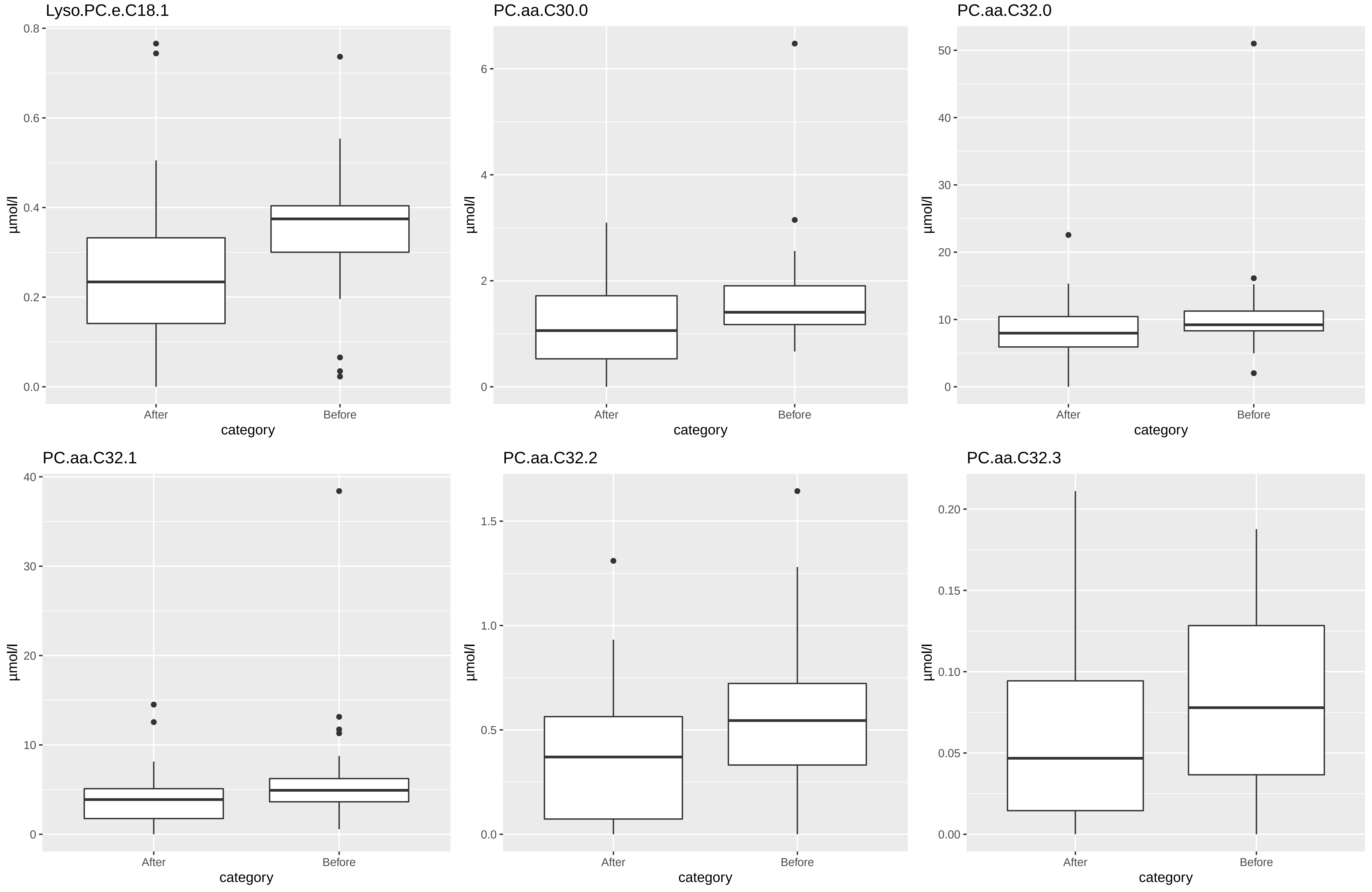
Lyso.PC.e.C18.1
PC.aa.C30.0
PC.aa.C32.0
PC.aa.C32.1
PC.aa.C32.2
PC.aa.C32.3
µmol/l
category
After
Before
0.0
0.2
0.4
0.6
0.8
0
2
4
6
10
20
30
40
50
0.5
1.0
1.5
0.00
0.05
0.10
0.15
0.20

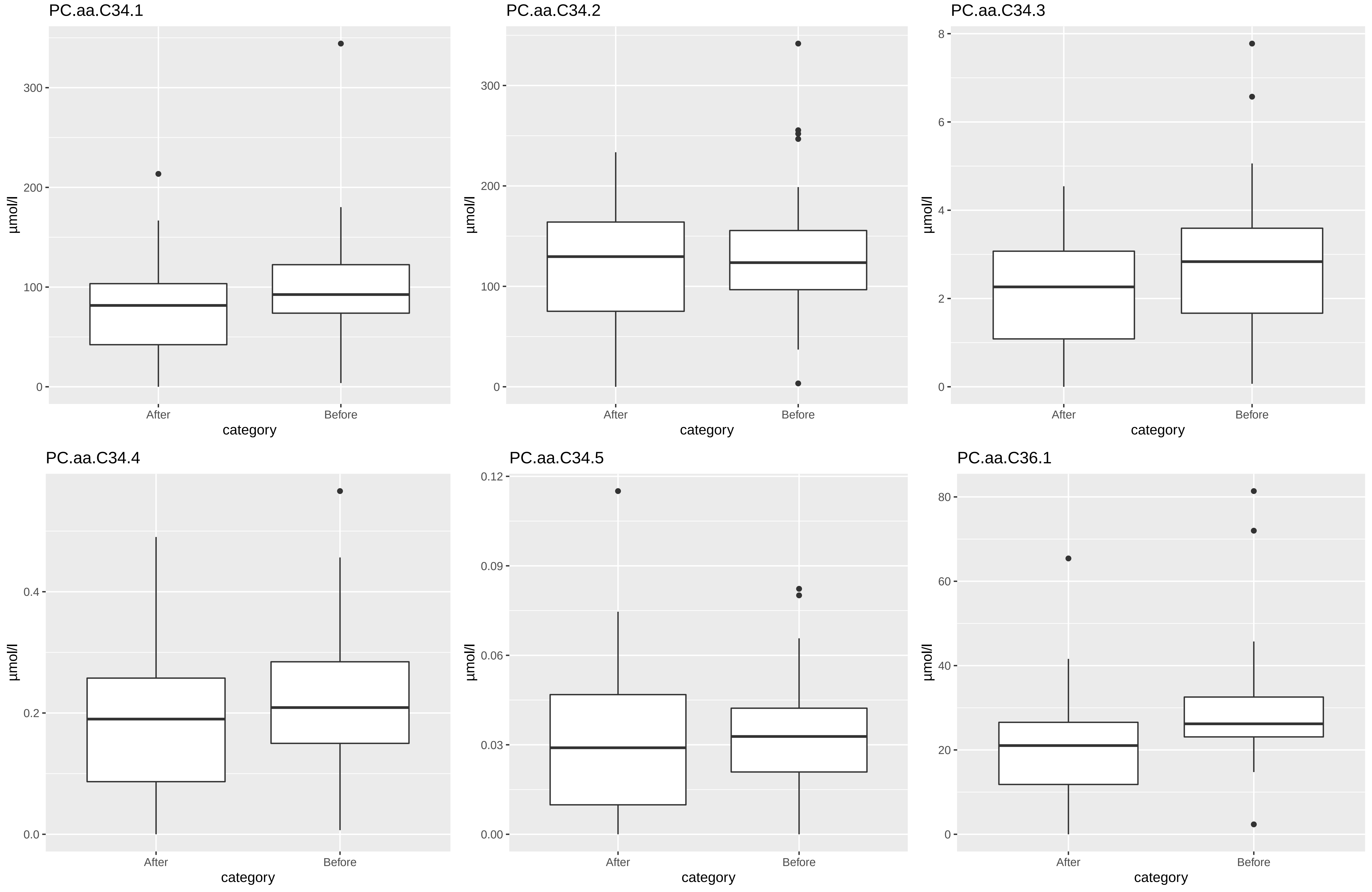

PC.aa.C34.1
PC.aa.C34.2
PC.aa.C34.3
PC.aa.C34.4
PC.aa.C34.5
PC.aa.C36.1
µmol/l
category
After
Before

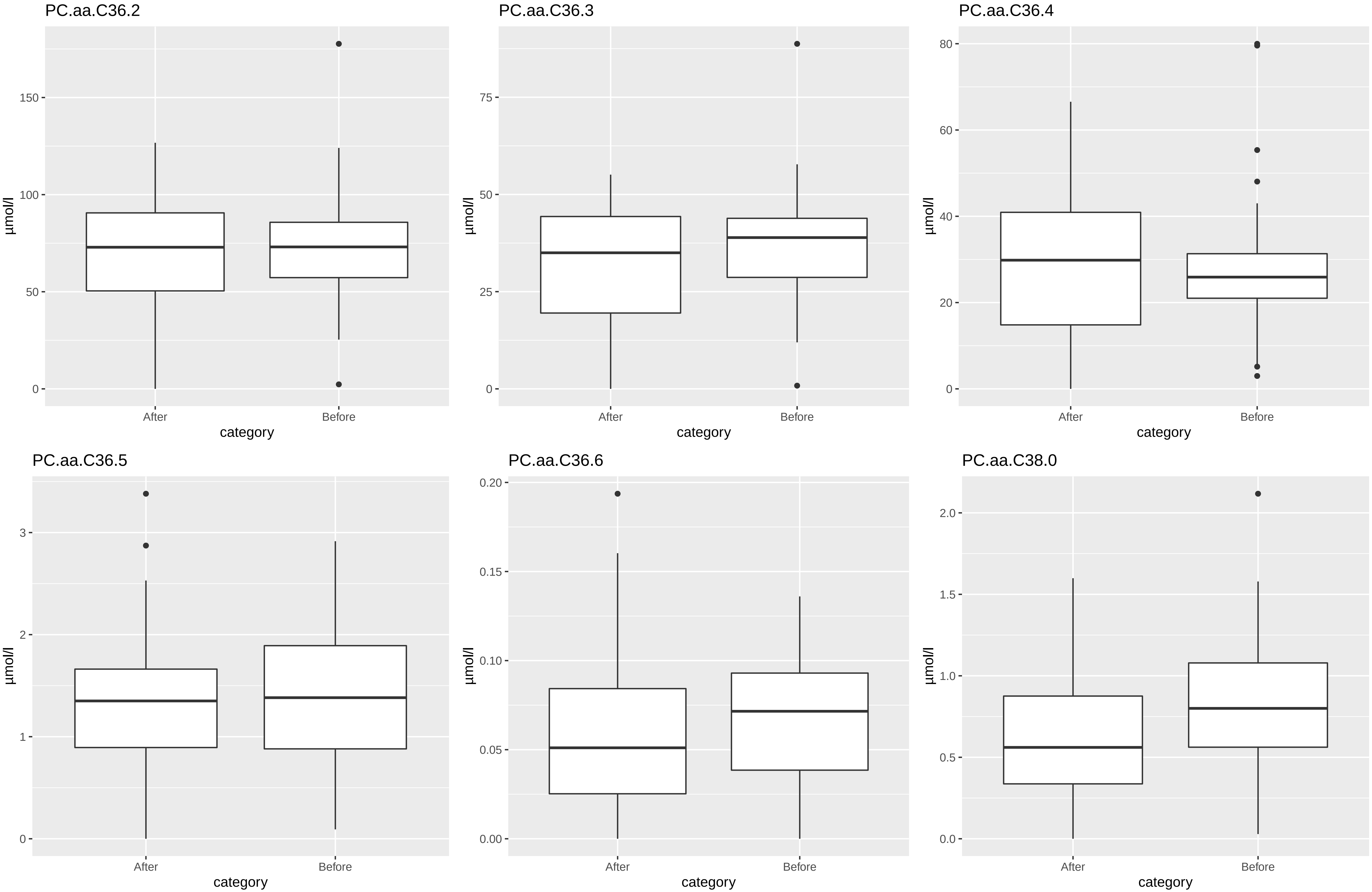
PC.aa.C36.2
PC.aa.C36.3
PC.aa.C36.4
PC.aa.C36.5
PC.aa.C36.6
PC.aa.C38.0
µmol/l
category
After
Before
0
50
100
150
25
75
20
40
60
80
1
2
3
0.00
0.05
0.10
0.15
0.20
0.0
0.5
1.0
1.5
2.0

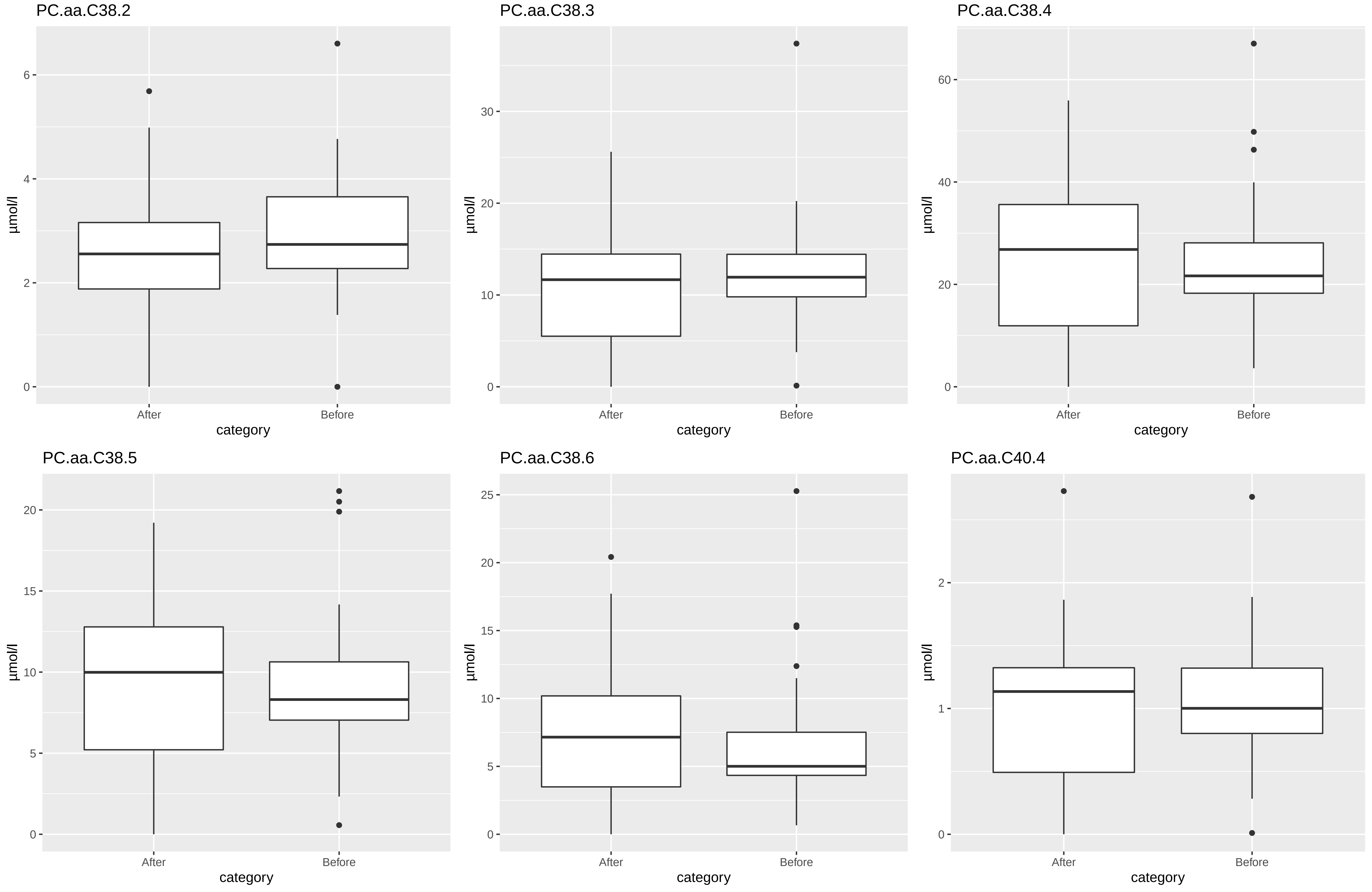
PC.aa.C38.2
PC.aa.C38.3
PC.aa.C38.4
PC.aa.C38.5
PC.aa.C38.6
PC.aa.C40.4
µmol/l
category
After
Before
0
2
4
6
10
20
30
40
60
5
15
25
1

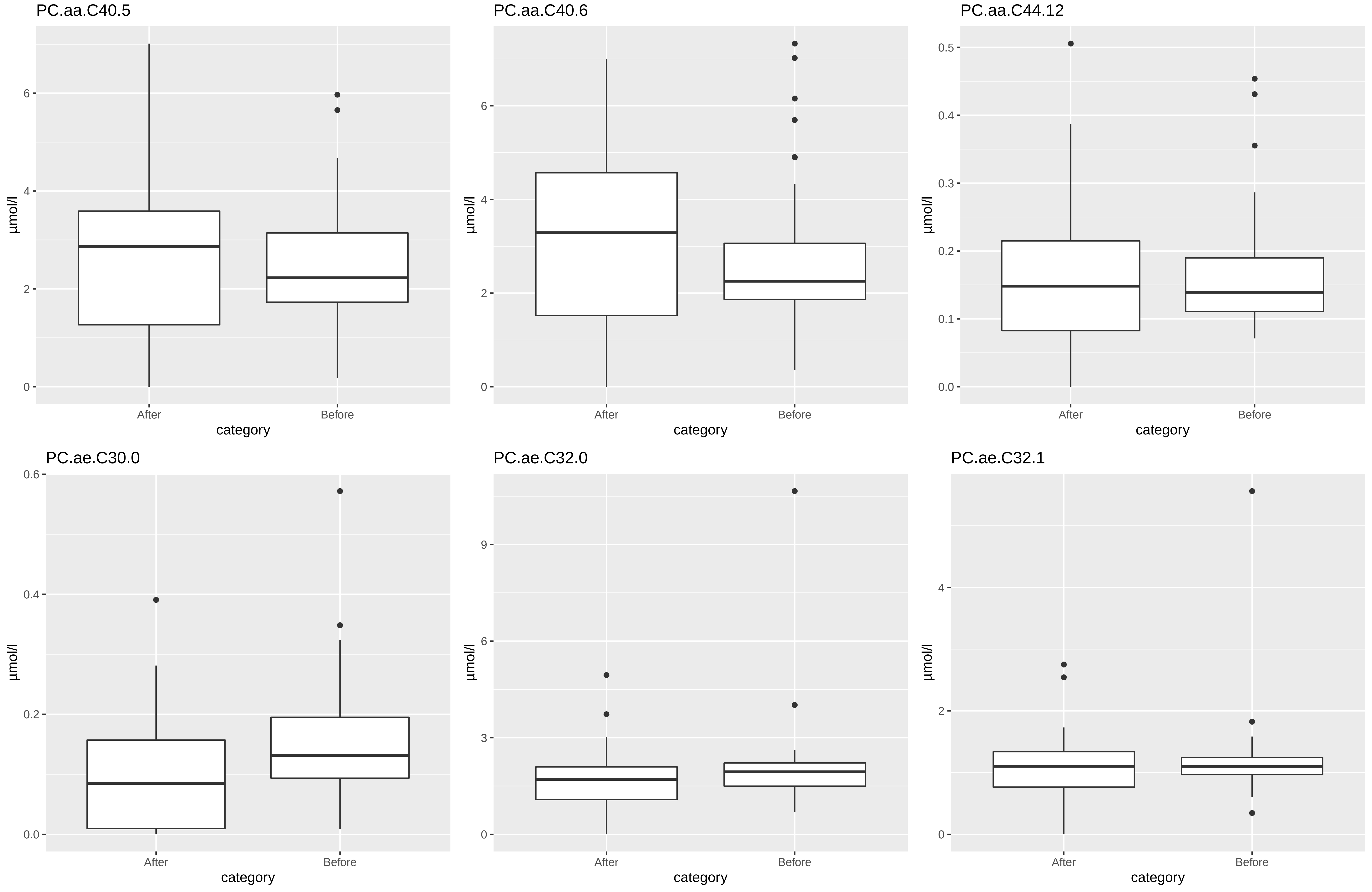
PC.aa.C40.5
PC.aa.C40.6
PC.aa.C44.12
PC.ae.C30.0
PC.ae.C32.0
PC.ae.C32.1
µmol/l
category
After
Before
0
2
4
6
0.0
0.1
0.2
0.3
0.4
0.5
0.6
3
9

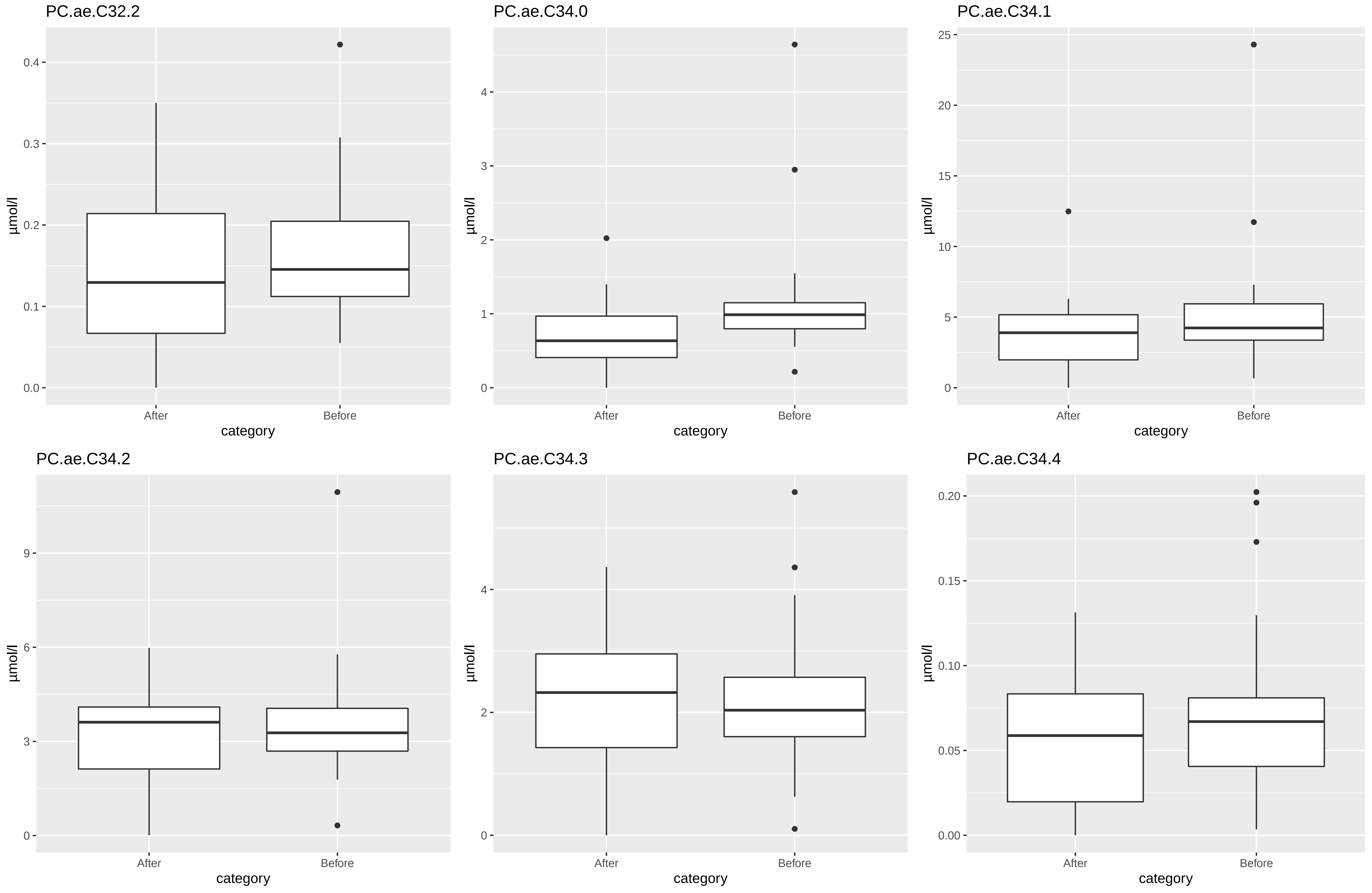
PC.ae.C32.2
0.4
0.3
0.2
0.1
0.0
µmol/l
After
Before
category
PC.ae.C34.0
4
3
2
1
0
µmol/l
After
Before
category
PC.ae.C34.1
25
20
15
10
5
0
µmol/l
After
Before
category
PC.ae.C34.2
9
6
3
0
µmol/l
After
Before
category
PC.ae.C34.3
4
2
0
µmol/l
After
Before
category
PC.ae.C34.4
0.20
0.15
0.10
0.05
0.00
µmol/l
After
Before
category

PC.ae.C36.0

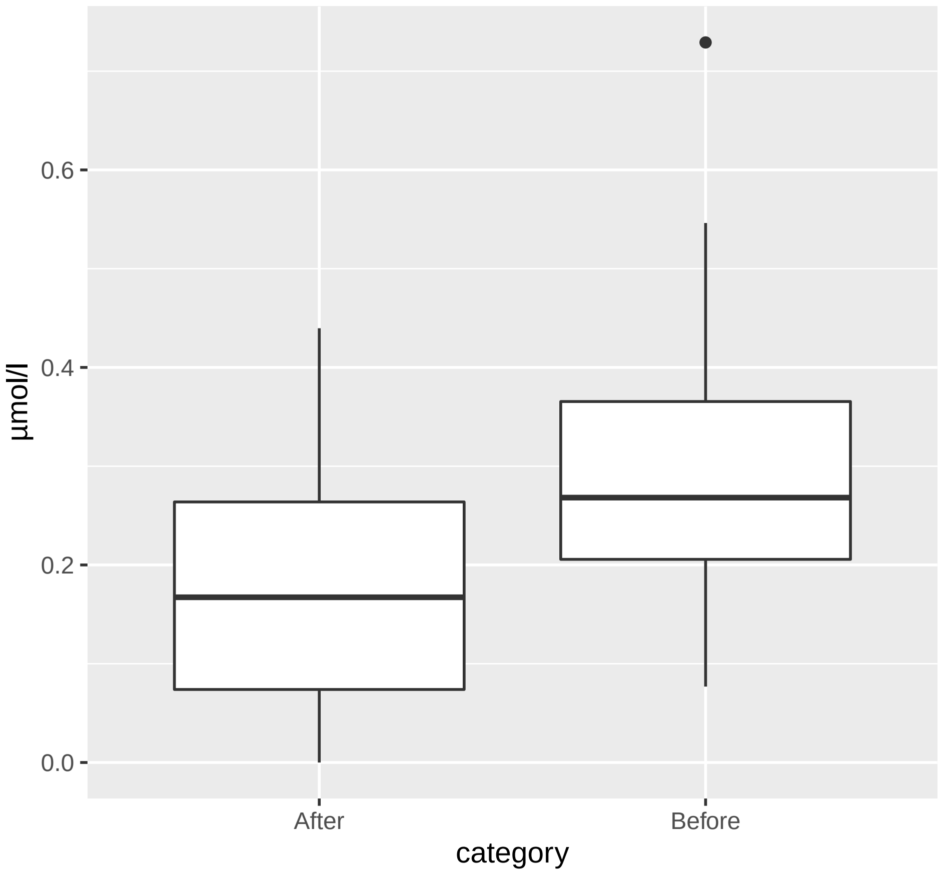

PC.ae.C36.1

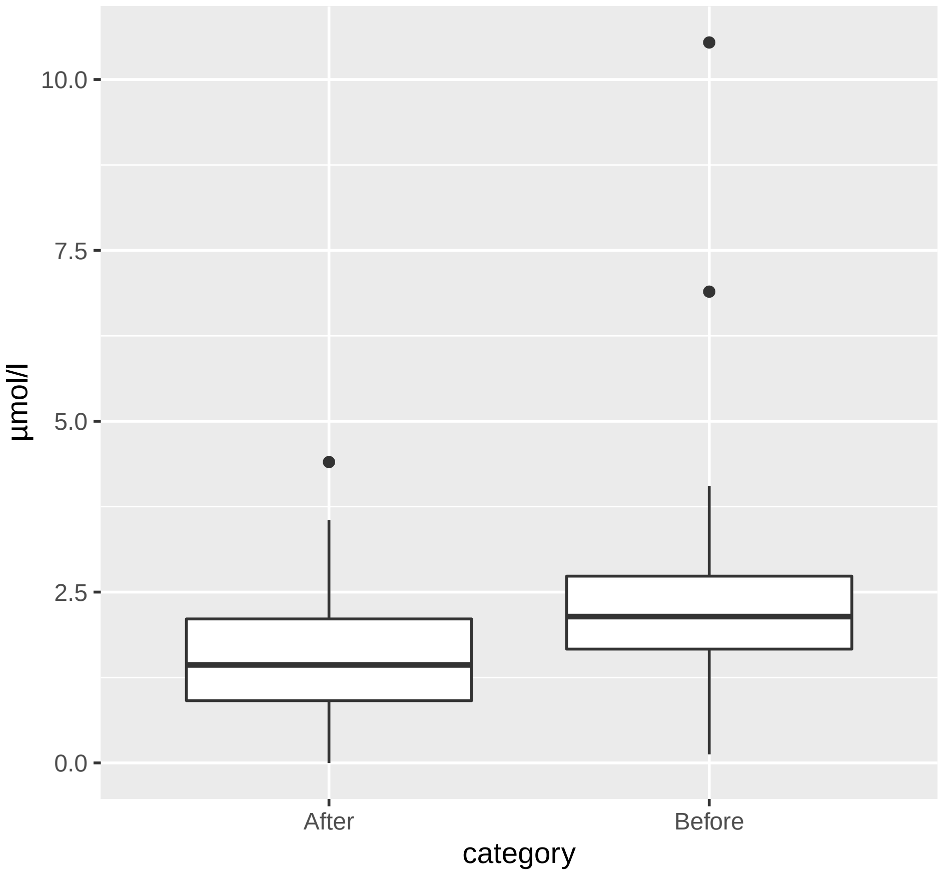

PC.ae.C36.2

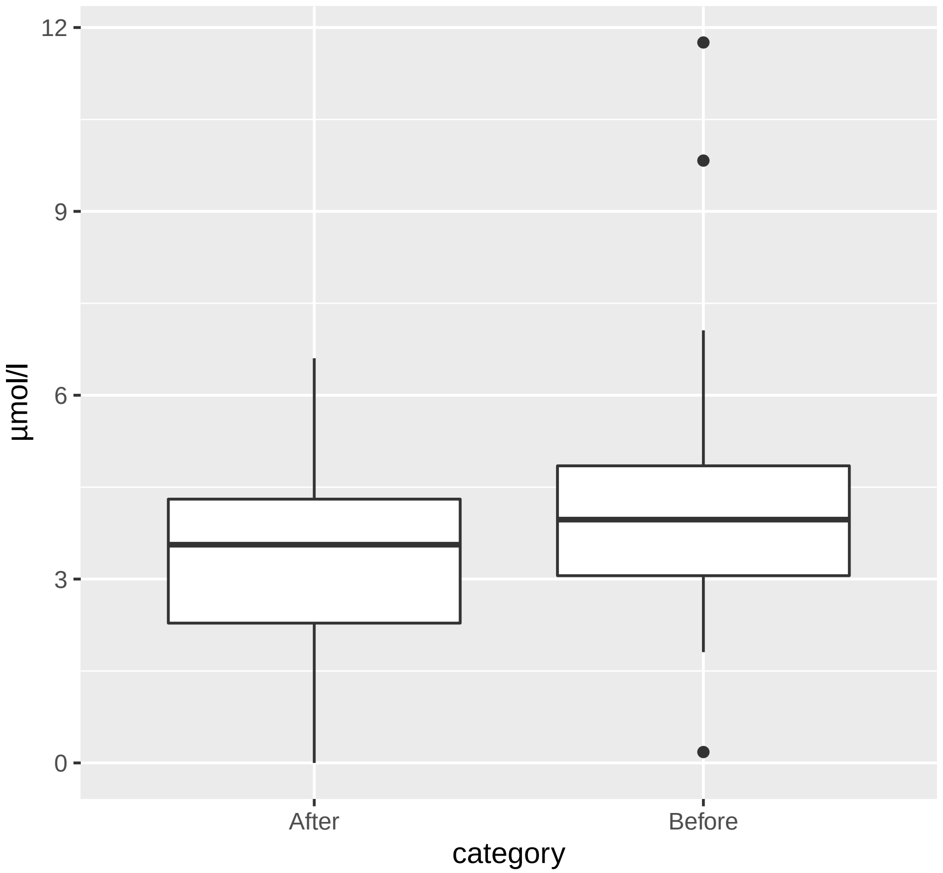

PC.ae.C36.3

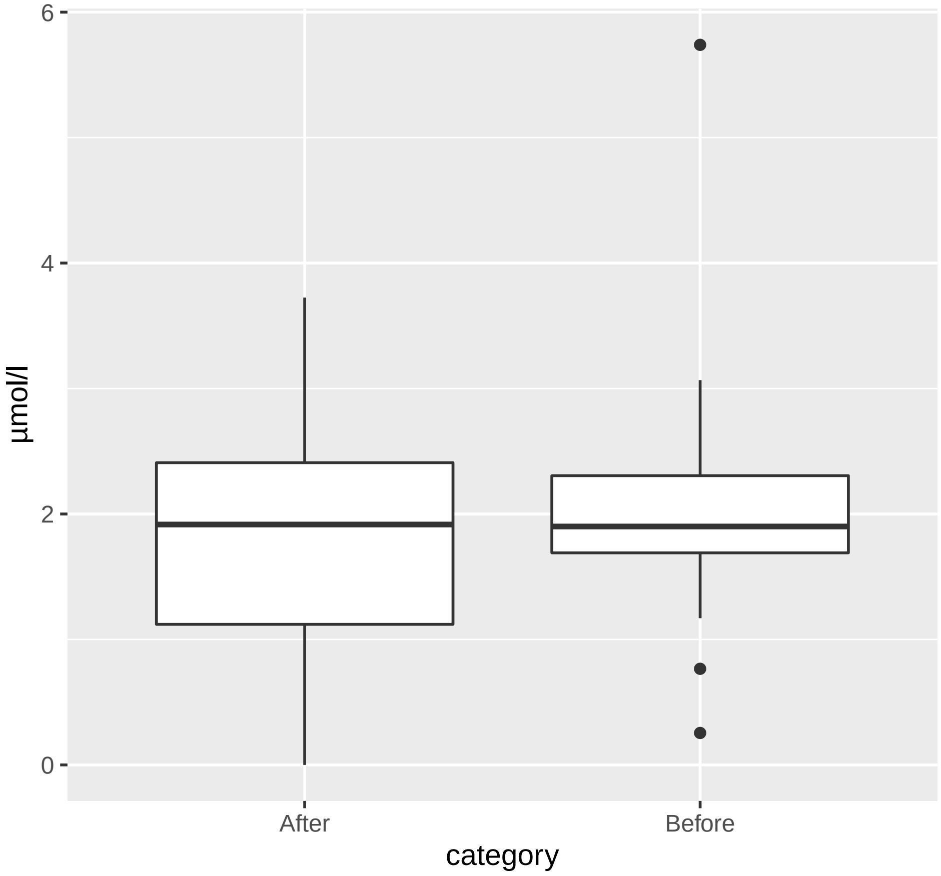

PC.ae.C36.4

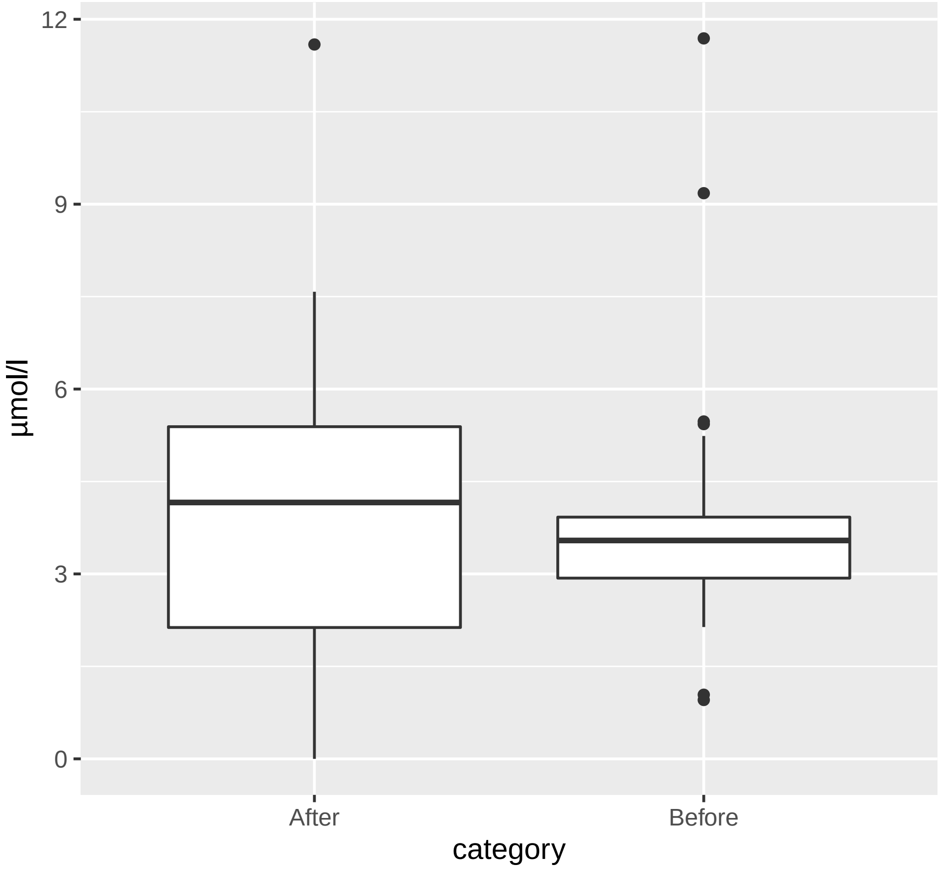

PC.ae.C36.5

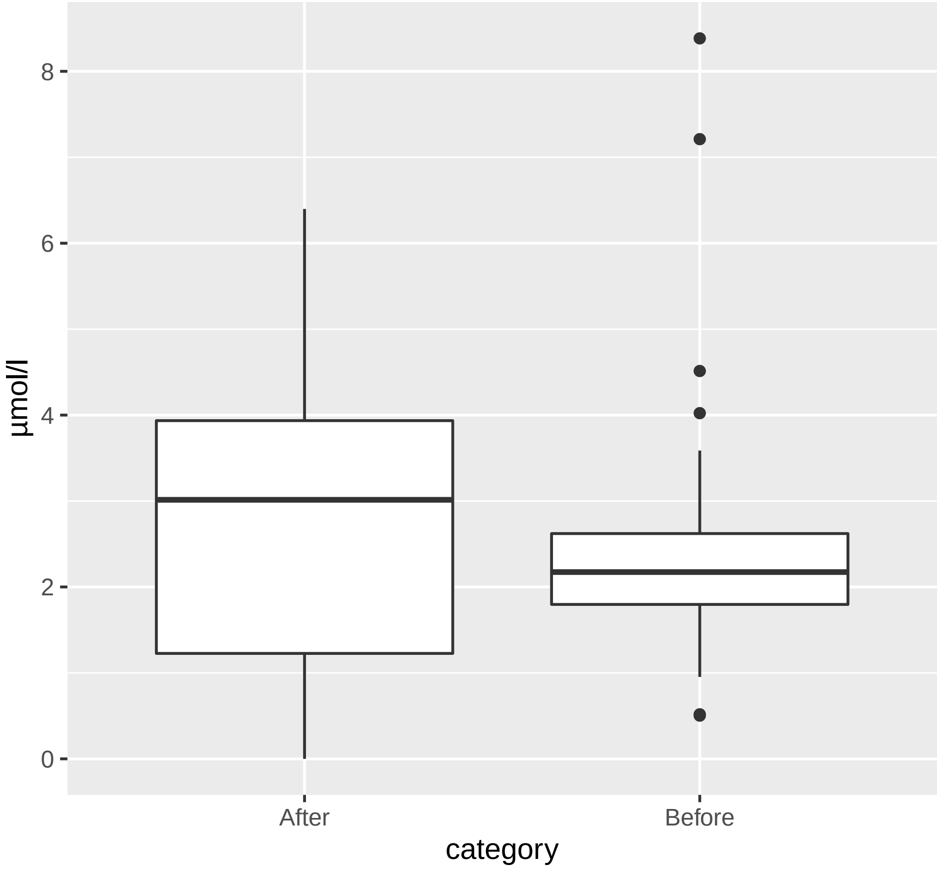

PC.ae.C36.6

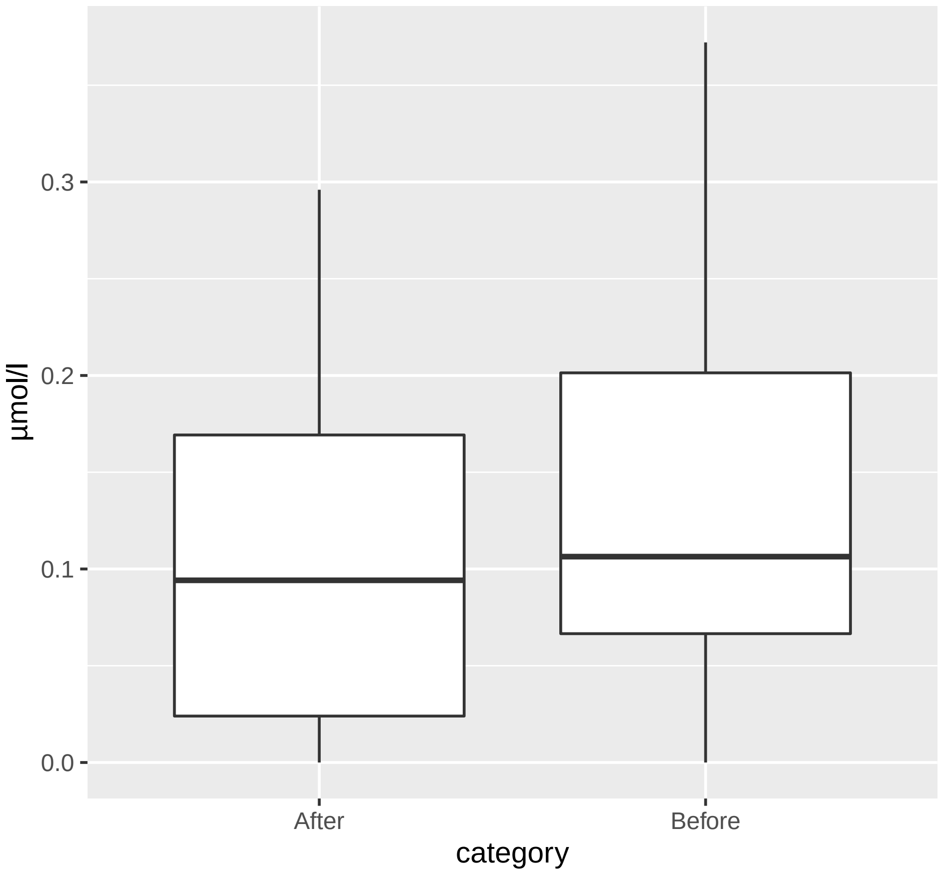

PC.ae.C38.0

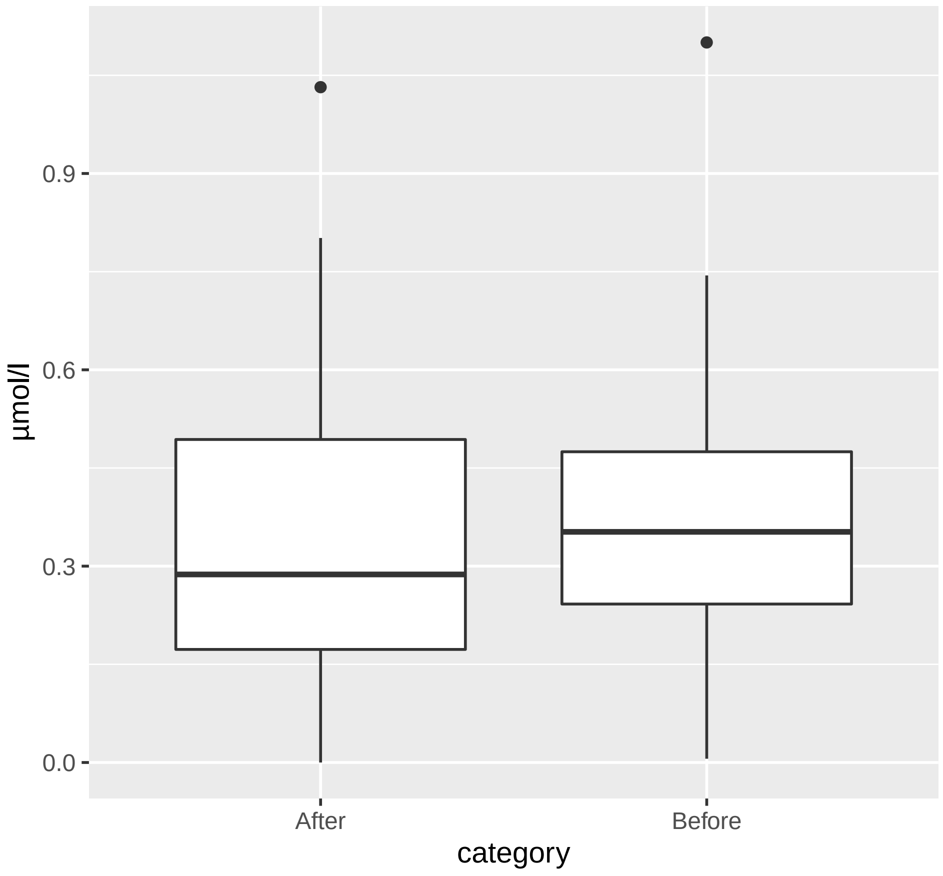

PC.ae.C38.3

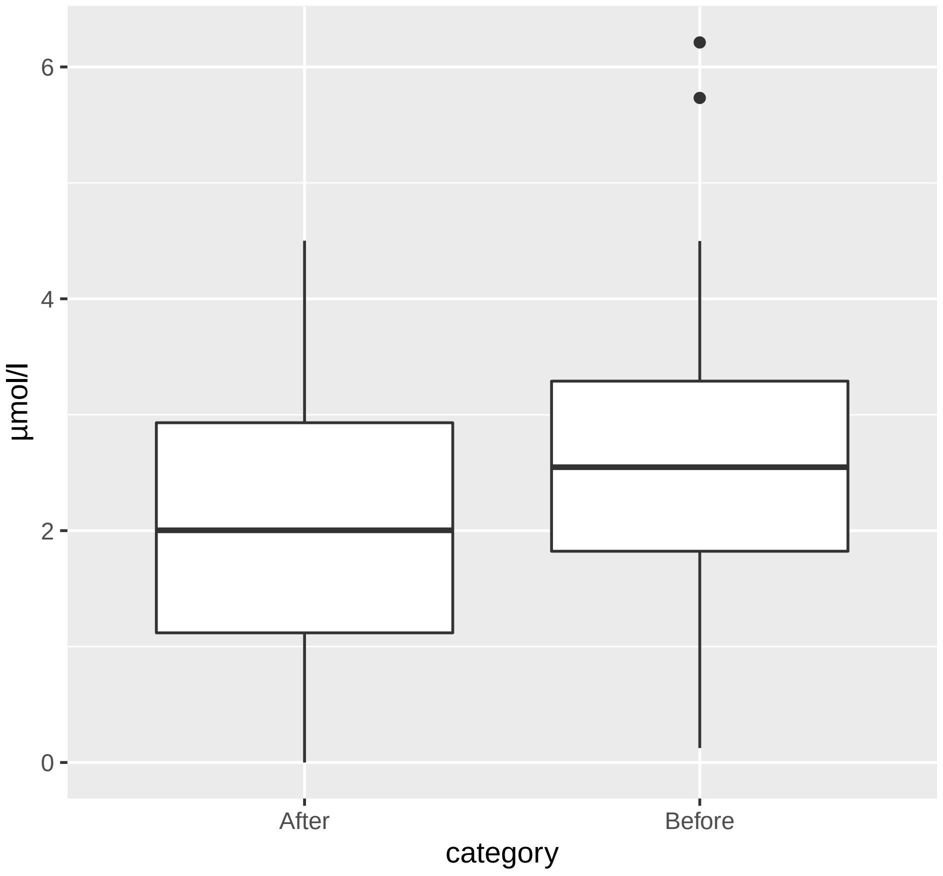

PC.ae.C38.4

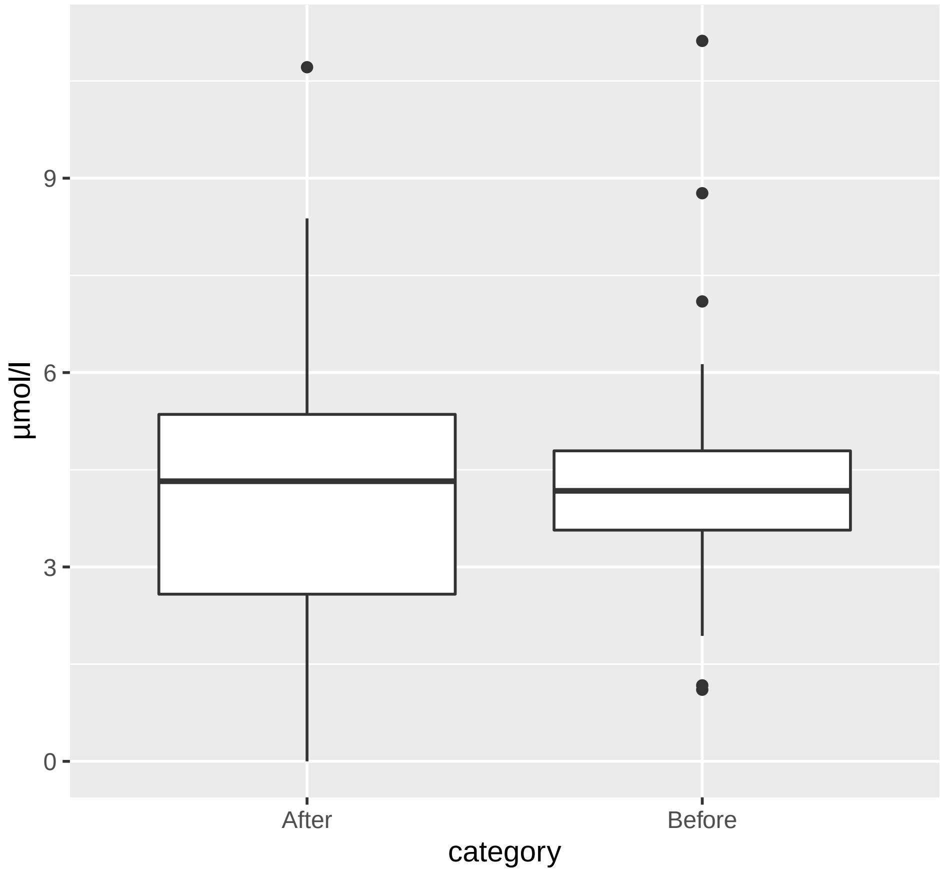

PC.ae.C38.5

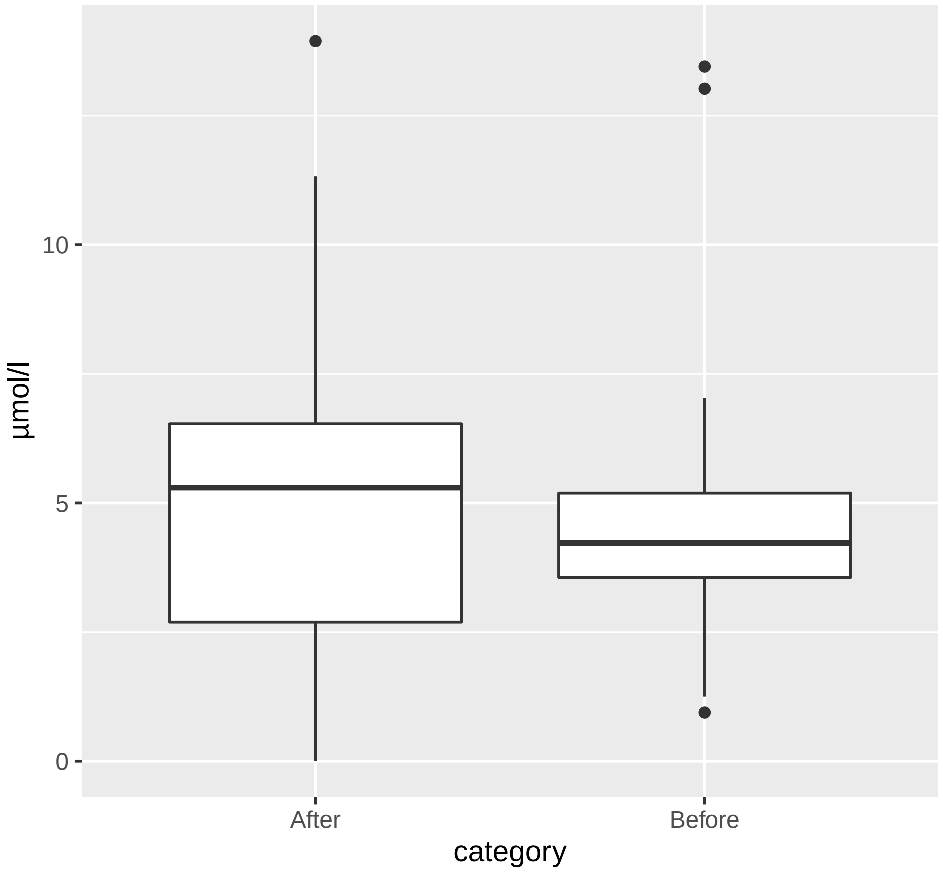

PC.ae.C38.6

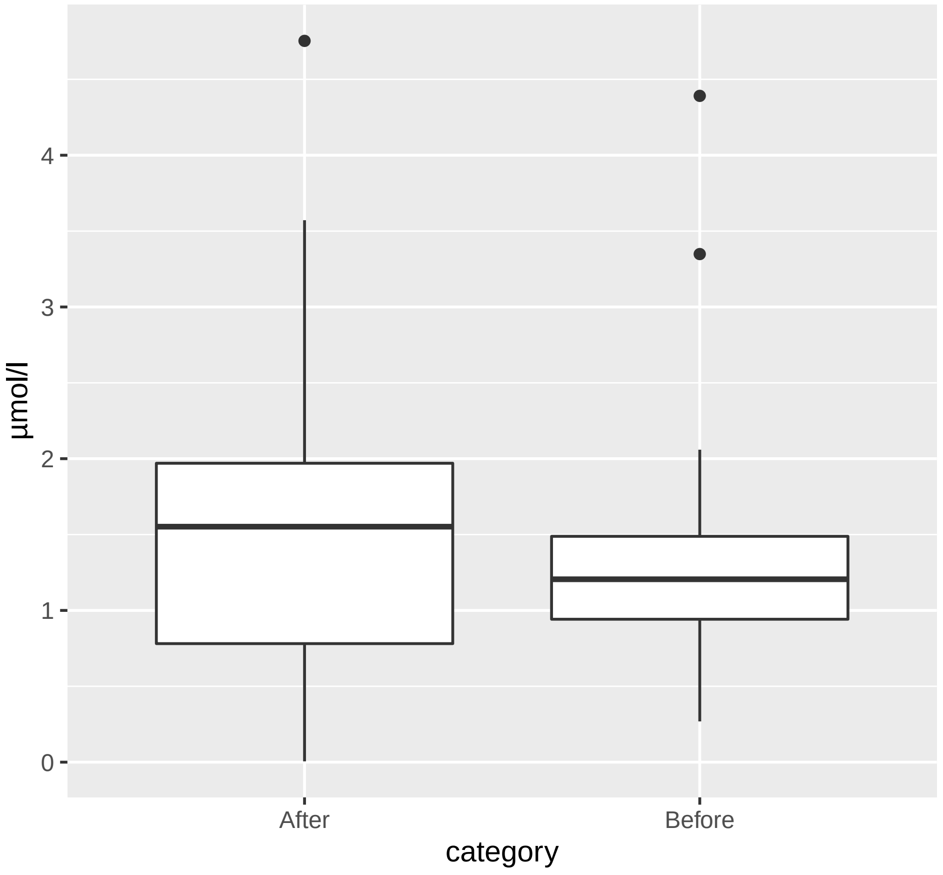

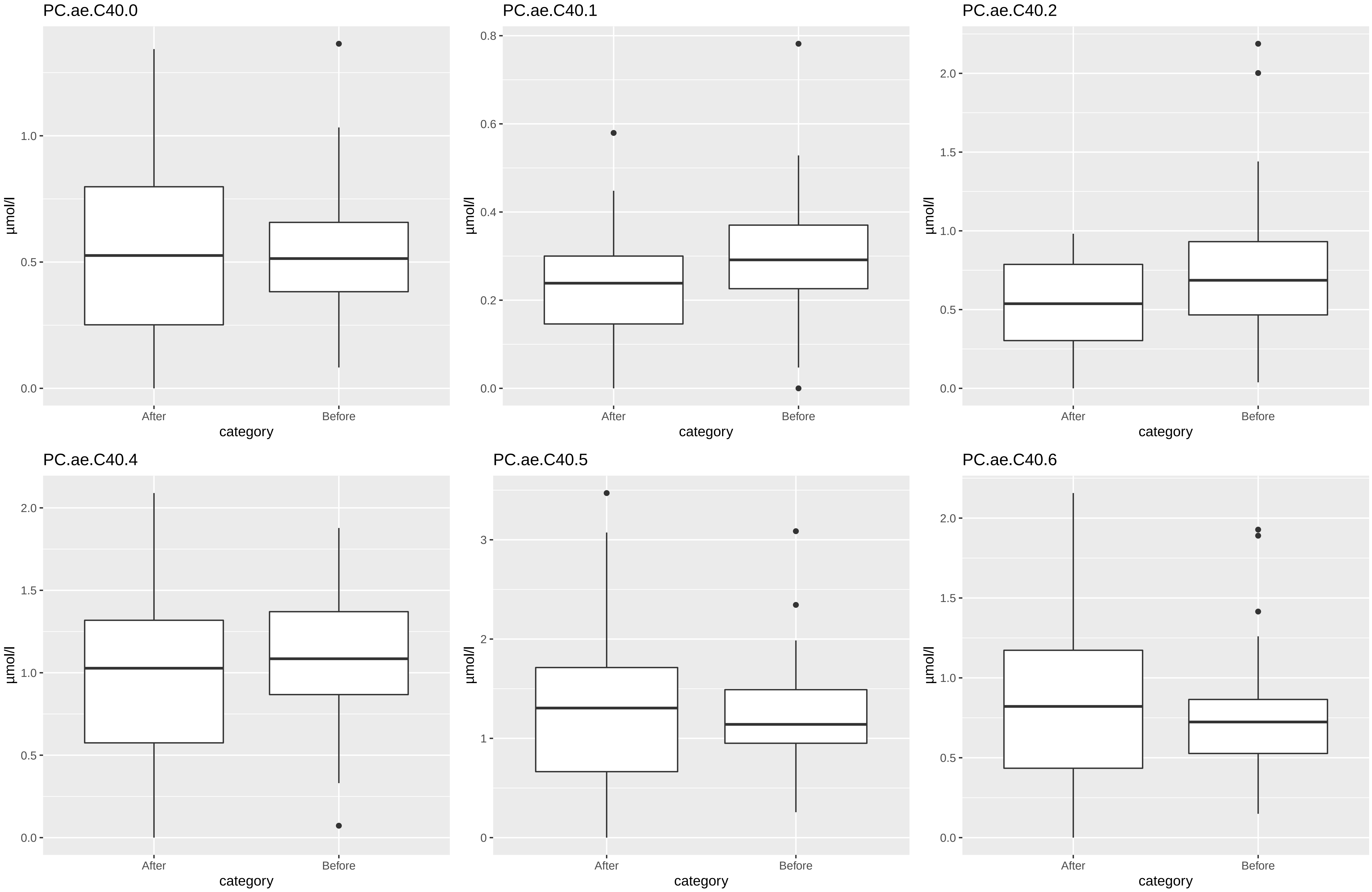
PC.ae.C40.0
PC.ae.C40.1
PC.ae.C40.2
PC.ae.C40.4
PC.ae.C40.5
PC.ae.C40.6
µmol/l
category
After
Before

PC.ae.C42.6

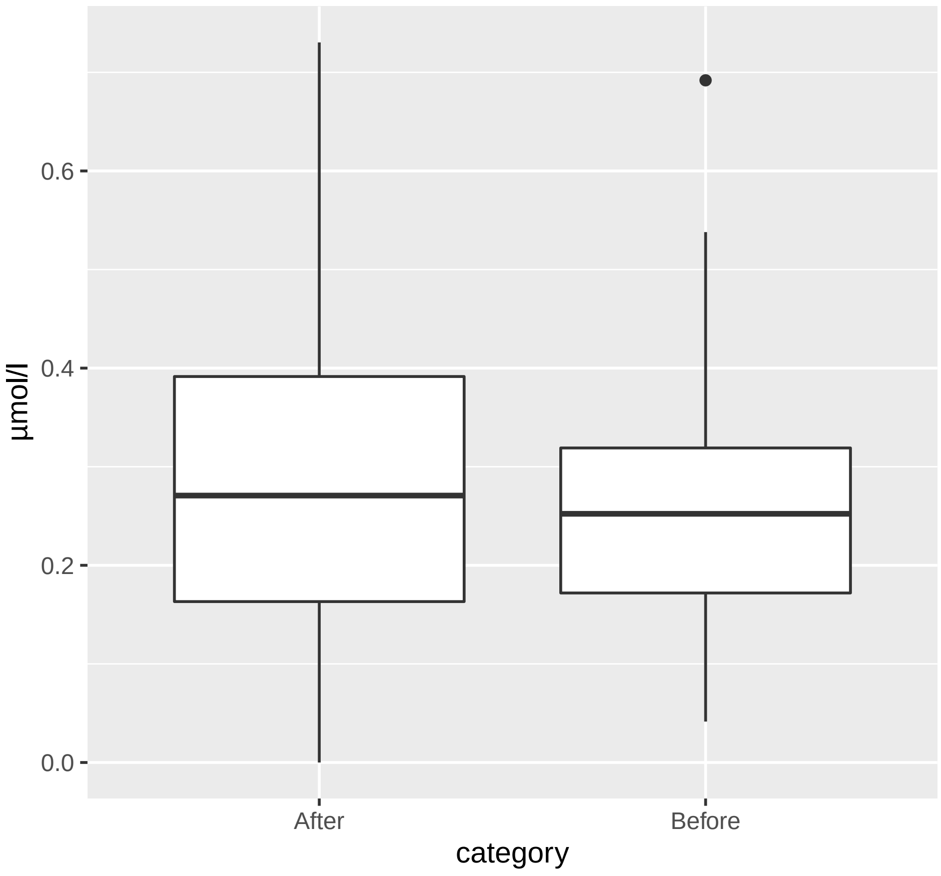

SM.a.C30.1

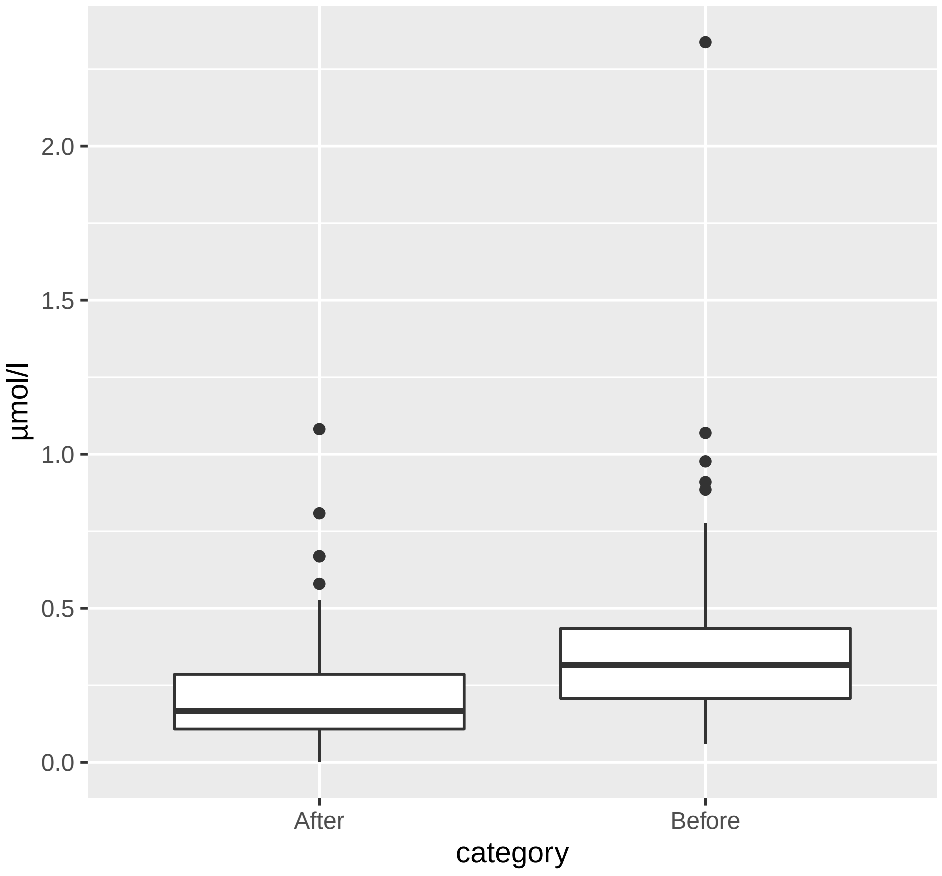

SM.a.C31.1

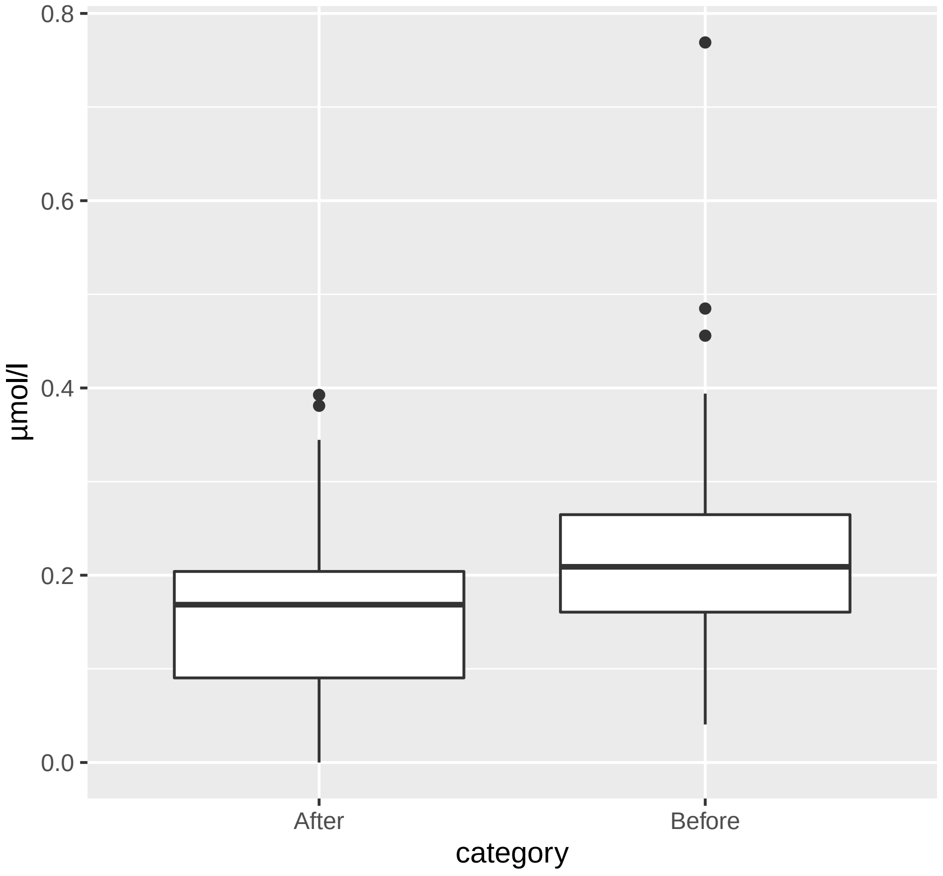

SM.a.C32.1

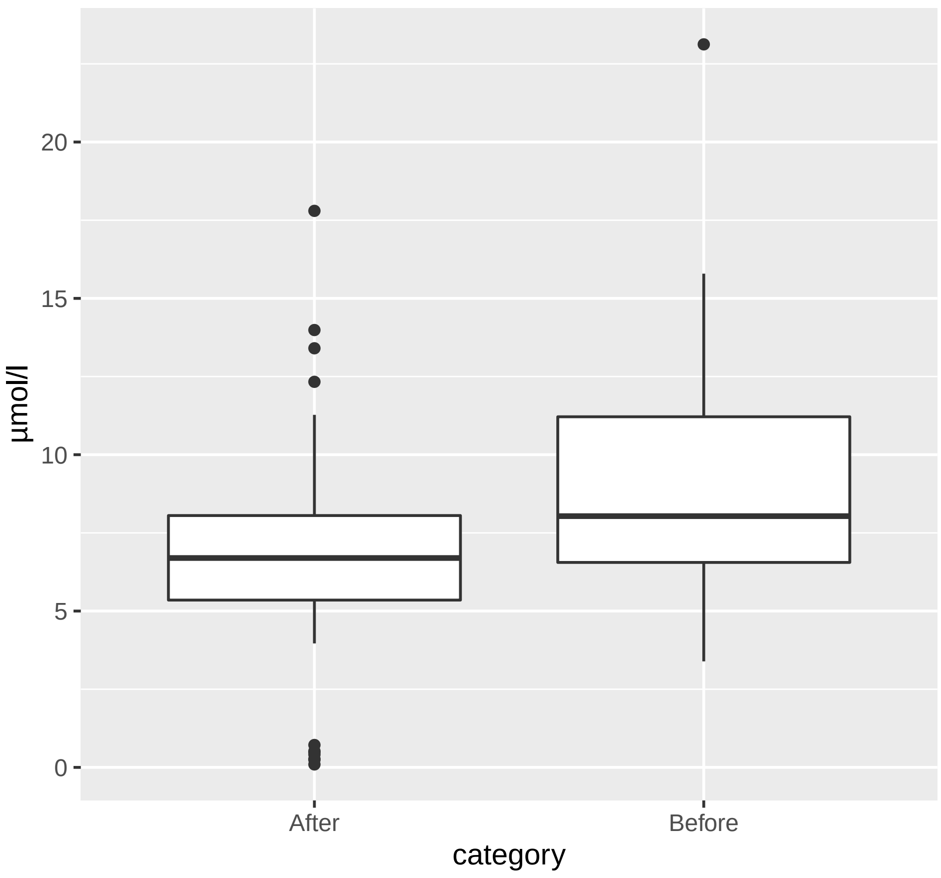

SM.a.C32.2

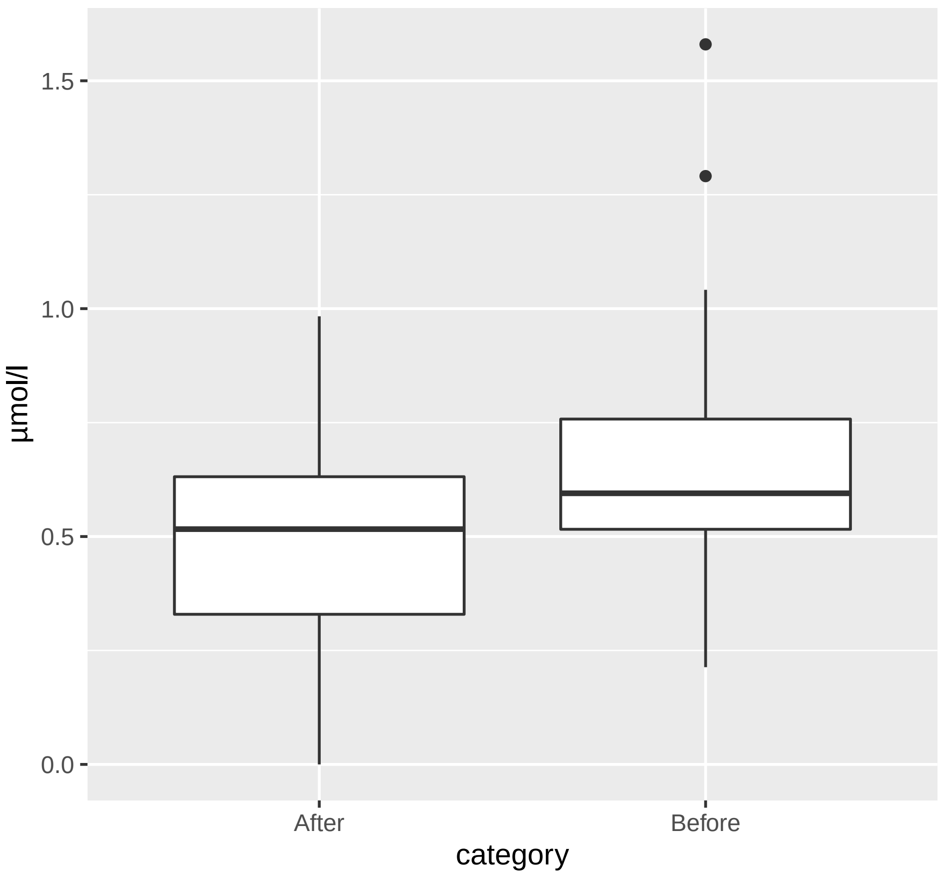

SM.a.C33.1

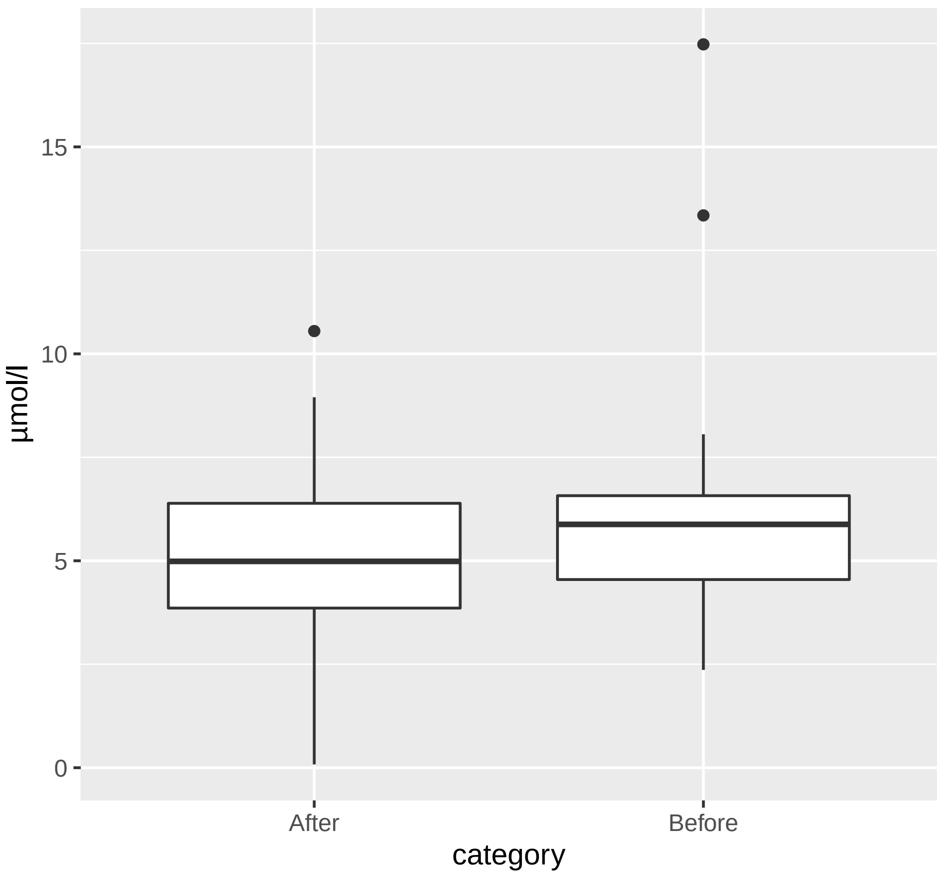

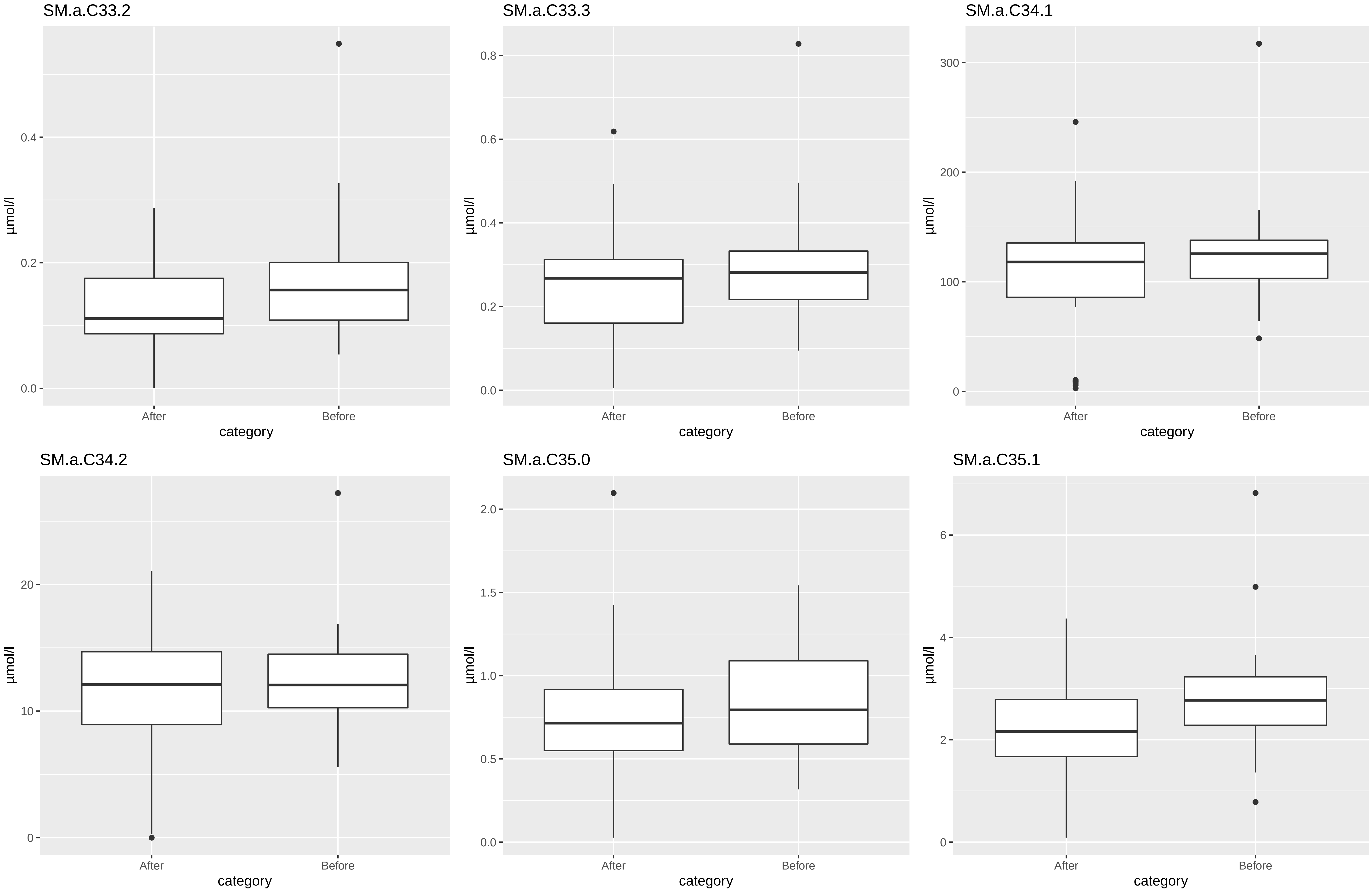
SM.a.C33.2
SM.a.C33.3
SM.a.C34.1
SM.a.C34.2
SM.a.C35.0
SM.a.C35.1
µmol/l
category
After
Before

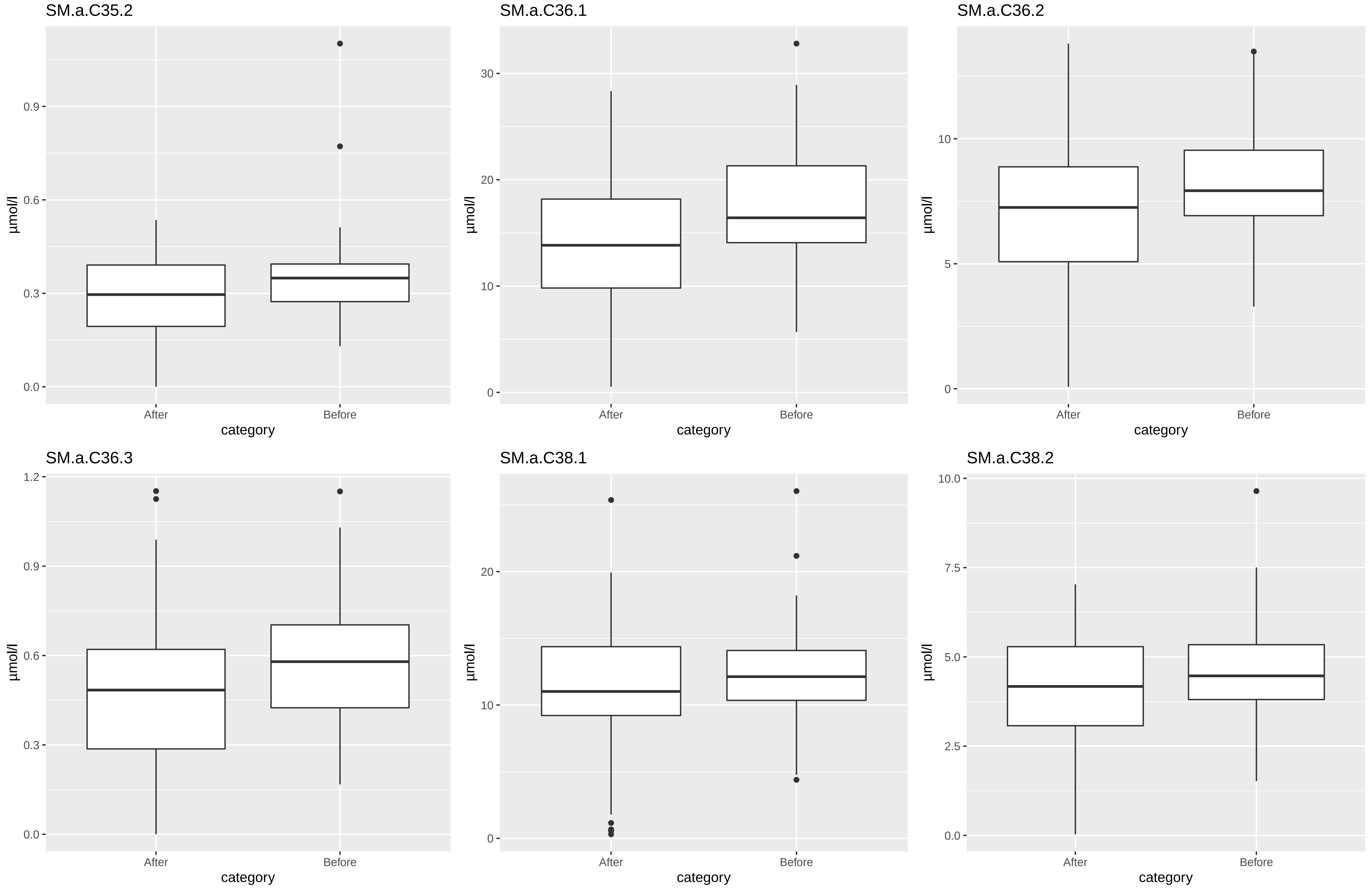
SM.a.C35.2
SM.a.C36.1
SM.a.C36.2
SM.a.C36.3
SM.a.C38.1
SM.a.C38.2
µmol/l
category
After
Before
0.0
0.3
0.6
0.9
1.2
0
5
10
20
30
2.5
5.0
7.5
10.0

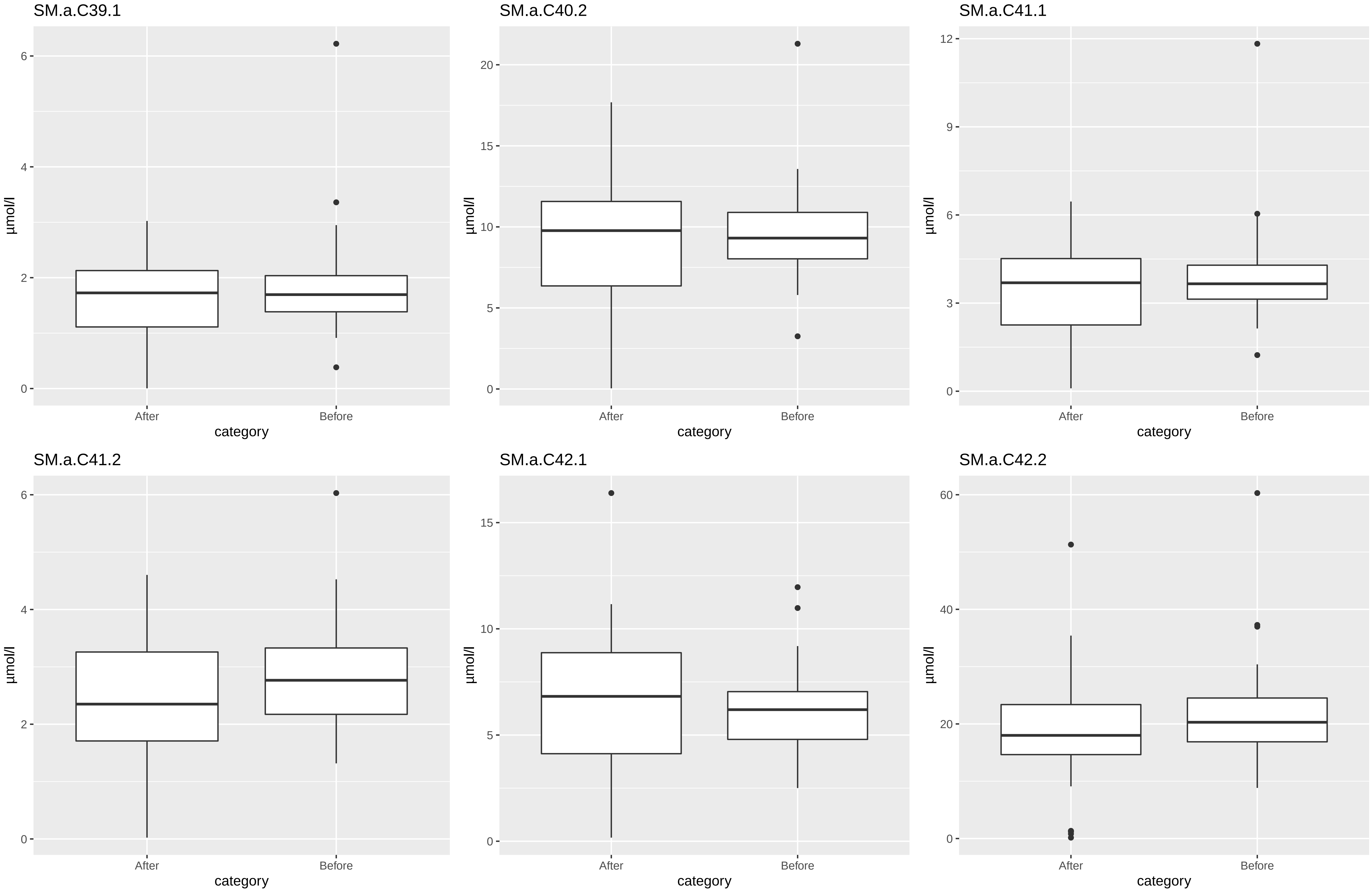
SM.a.C39.1
SM.a.C40.2
SM.a.C41.1
SM.a.C41.2
SM.a.C42.1
SM.a.C42.2
µmol/l
category
After
Before

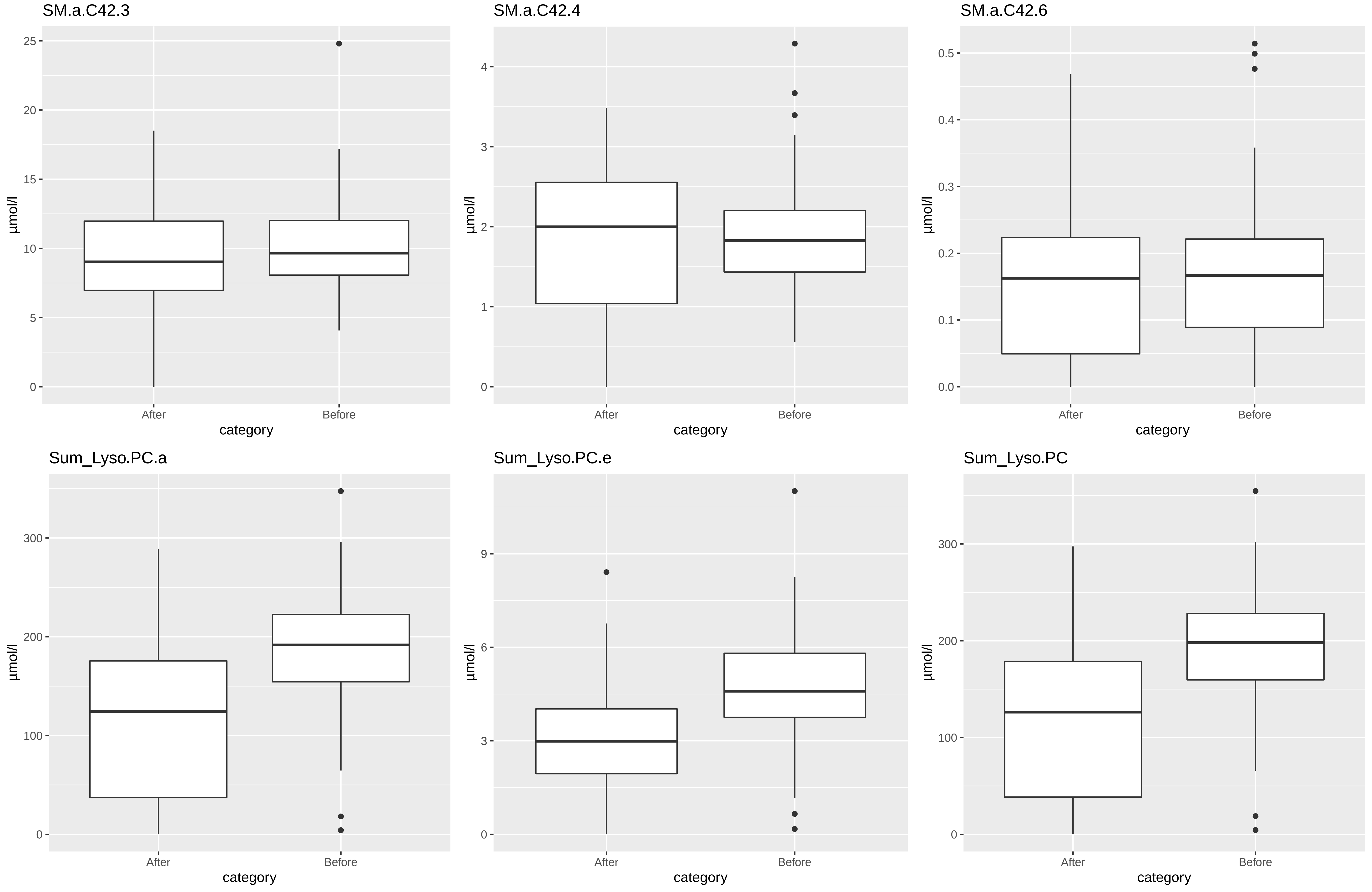
SM.a.C42.3
SM.a.C42.4
SM.a.C42.6
Sum_Lyso.PC.a
Sum_Lyso.PC.e
Sum_Lyso.PC
µmol/l
category
After
Before

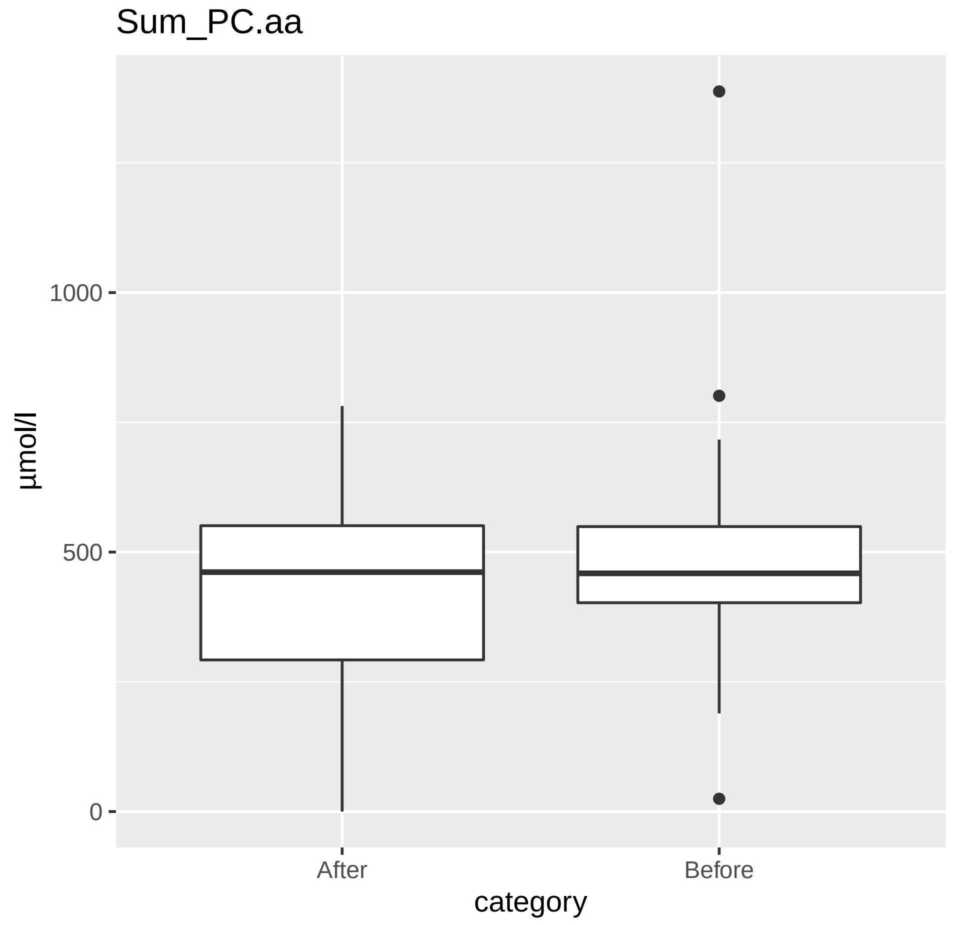
Sum_PC.aa
µmol/l
0
500
1000
After
Before
category

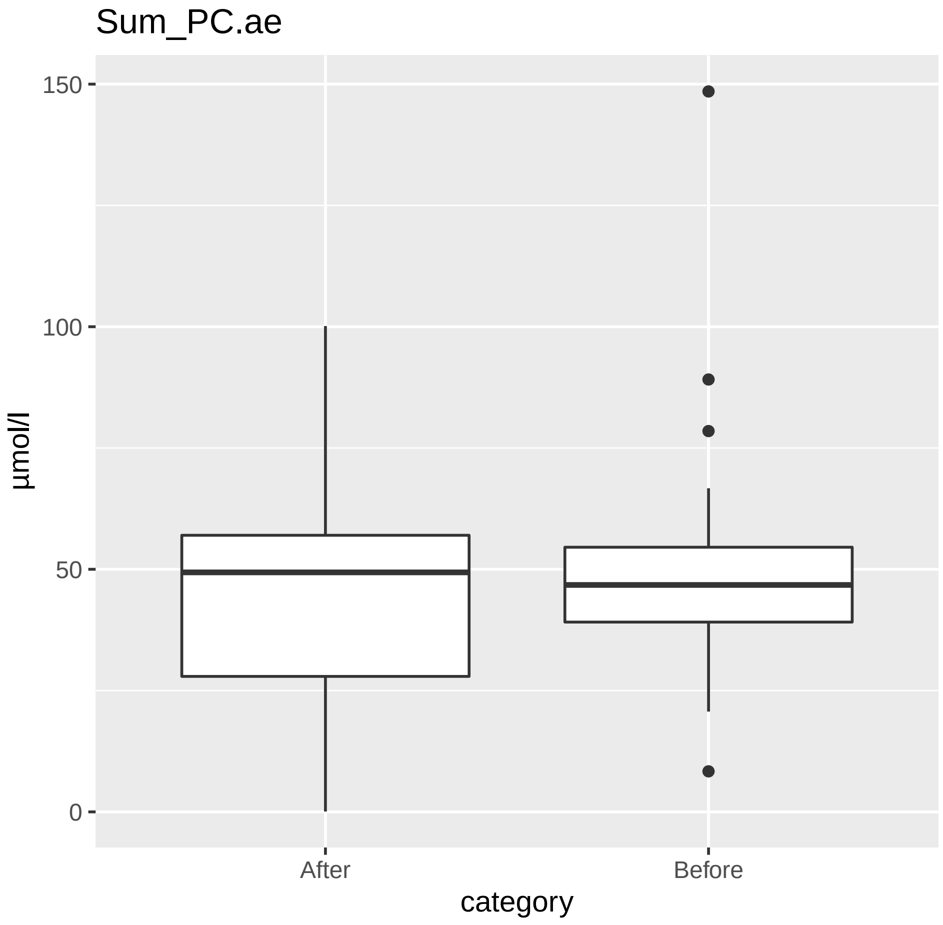
Sum_PC.ae
µmol/l
0
50
100
150
After
Before
category

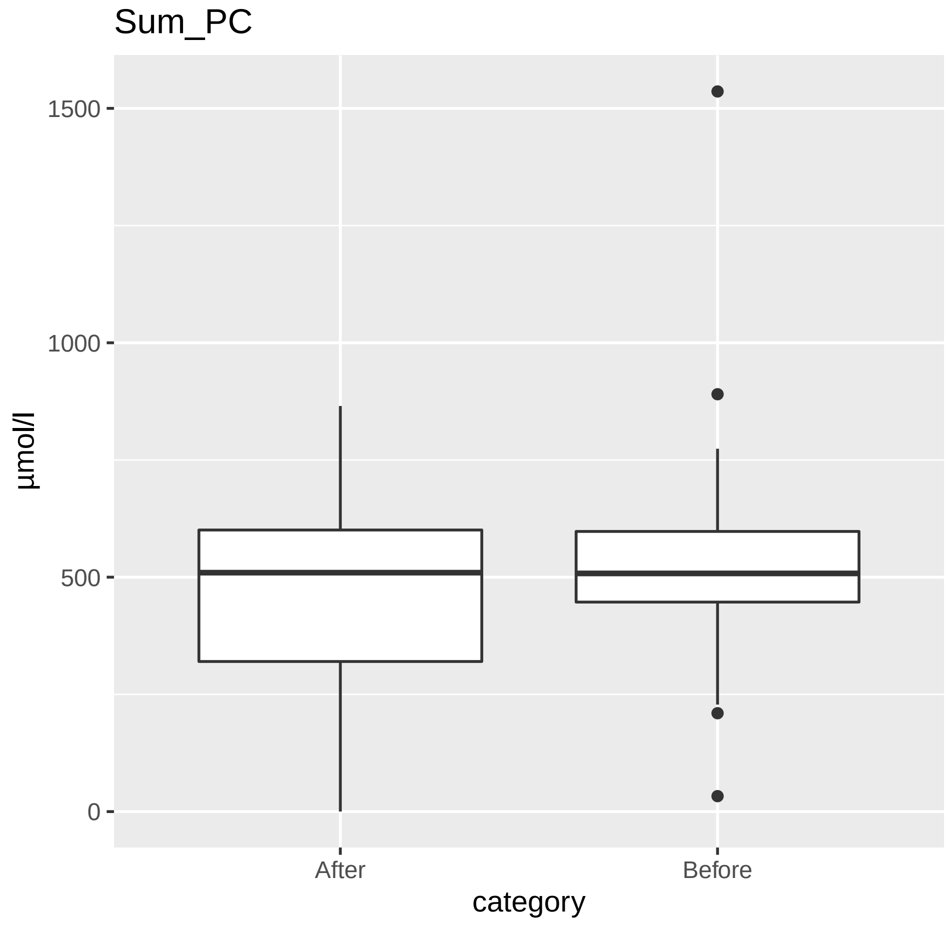
Sum_PC
µmol/l
0
500
1000
1500
After
Before
category

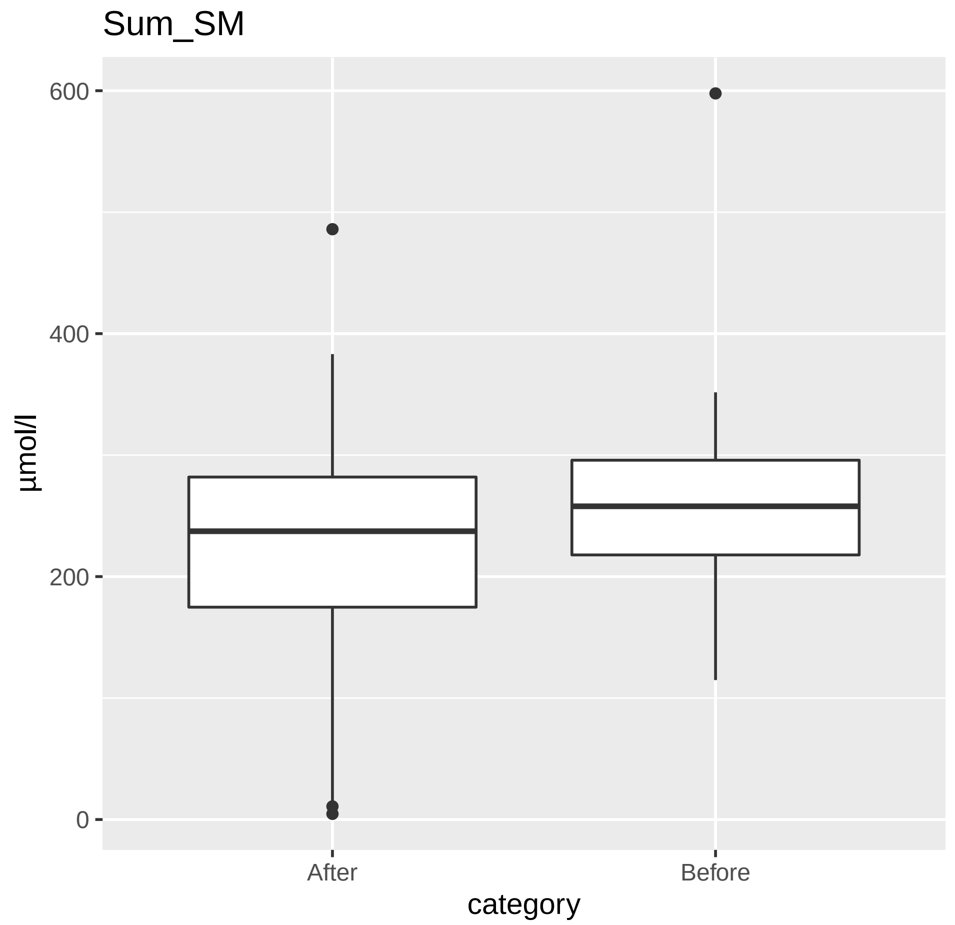
Sum_SM
µmol/l
0
200
400
600
After
Before
category
